# Porphyrin pathways as targets for combating antimicrobial resistance: virulence and therapeutic opportunities

**DOI:** 10.3389/fcimb.2026.1842791

**Published:** 2026-07-13

**Authors:** Alysha Pyne, Jameel M. Abduljalil, Ann H. Kwan

**Affiliations:** 1 School of Life and Environmental Sciences, University of Sydney, Sydney, NSW, Australia; 2 The SydneyNano Institute, University of Sydney, Sydney, NSW, Australia

**Keywords:** antimicrobial development, antimicrobial resistance, bacterial virulence, heme biology, host-pathogen interactions, porphyrin biology

## Abstract

Porphyrins are versatile biomolecules central to the synthesis of many essential molecules across all domains of life. Their ability to absorb light, accept electrons and facilitate redox reactions underpins diverse biological roles. Importantly, porphyrin properties can be easily tailored through substitutions of side chains and chelated metals. This adaptability has enabled porphyrins to play critical roles in bacterial environmental adaptations, most notably in enhancing competitive advantage and virulence. The association between porphyrin adaptations and virulence in pathogenic bacteria presents a unique opportunity for the development of next-generation antibacterial strategies and therapeutics. By focusing on virulence and virulence-related systems, these innovative strategies aim to address antimicrobial resistance by reducing the selective pressures that drive its emergence, promote evolutionary pathways that favor non-pathogenic adaptations and help to restore the balance of commensal microbiota. This review highlights the recent advances in porphyrin and porphyrin-related antibiotic developments, with emphasis on approaches targeting virulence versus bacterial survival. We further discuss emerging virulence-targeted therapeutics, including artificial intelligence and machine learning for novel target and lead discovery.

## 1 Introduction

The alarming escalation of antimicrobial resistance (AMR) presents a considerable risk to global public health and the stability of modern medical practices. Once-treatable infections are increasingly associated with poor clinical outcomes, with recent estimates attributing approximately 1.3 million deaths to AMR in 2019 across 204 countries, with projected tolls of 10 million deaths worldwide by 2050 (World Health Organisation, 2014; Murray et al., 2022). Current mitigation efforts focus largely on antibiotic stewardship in clinical and agricultural settings, including restricting broad-spectrum antibiotic use, prioritizing narrow-spectrum agents, and minimizing unnecessary prescriptions (Prestinaci et al., 2015). Although new antimicrobials and combination therapies continue to emerge, these approaches primarily slow resistance rather than eliminate the selective pressures that drive it, leaving a persistent need for new therapeutic strategies (Prestinaci et al., 2015).

One promising direction is the development of therapeutics that target bacterial virulence rather than viability. By disarming pathogens instead of eradicating them, virulence-directed therapeutics may reduce the evolutionary incentives that favor resistance while promoting bacterial adaptations compatible with commensal or non-pathogenic lifestyles. Such strategies require targets that are central to pathogenic fitness but not essential for basal survival. Porphyrins and porphyrin-centered metabolic pathways represent highly suitable targets for such approaches as they lie at the intersection of bacterial metabolism, stress tolerance, virulence, and host-pathogen interactions.

**Figure 1:**
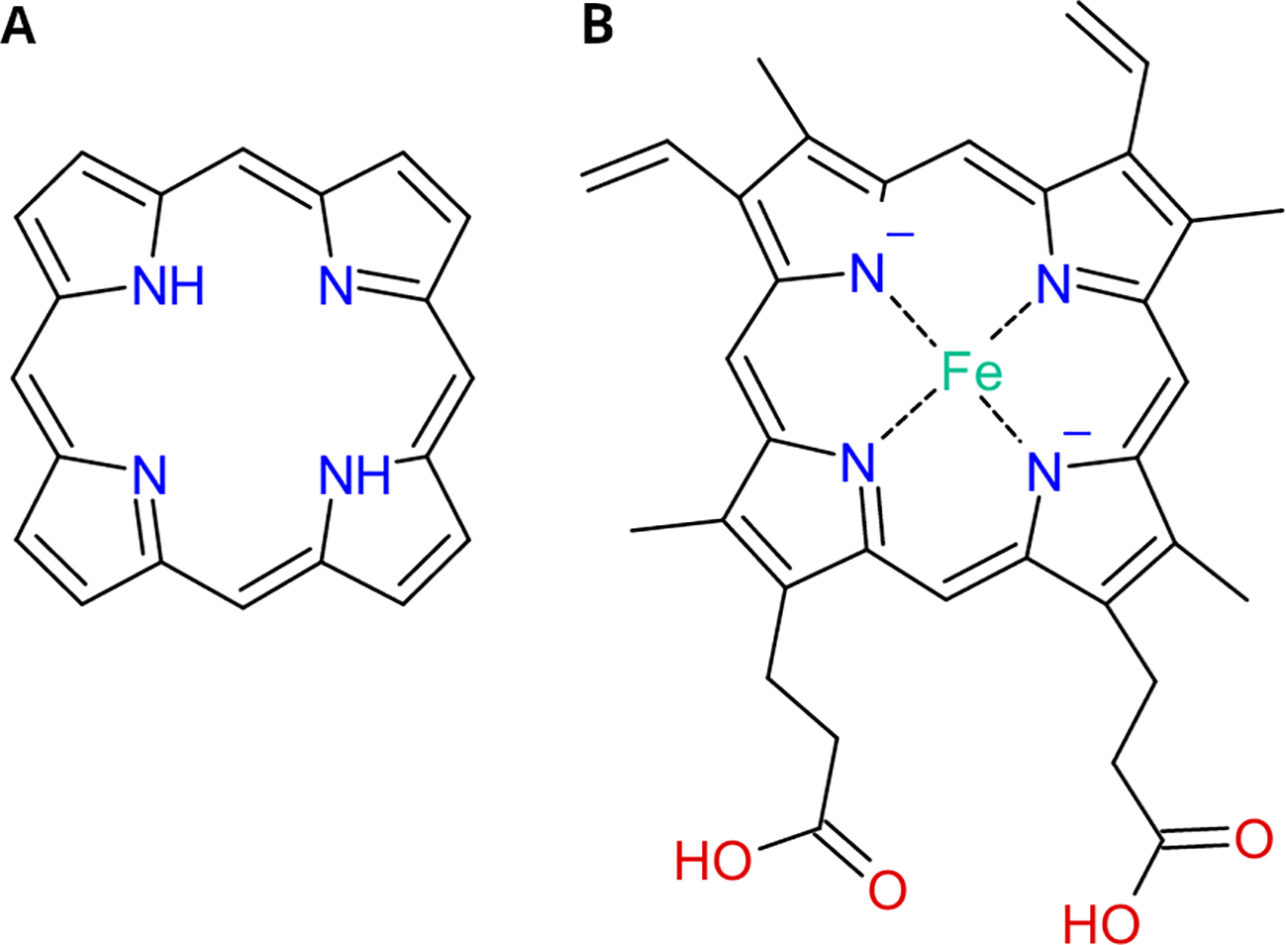
General chemical structure of porphyrins and heme. The basic structures of **(A)** the core porphyrin tetrapyrrole ring, and **(B)** the iron-containing protoporphyrin IX, FePPIX, heme.

Porphyrins are macromolecules used in the biosynthesis of many essential molecules critical to numerous essential biological processes, including respiration, photosynthesis, a wide variety of sensor-based and signal transduction pathways, and diverse redox-driven enzymatic reactions (Shimizu et al., 2019; Tanaka and Tanaka, 2007; Rowley and Kendall, 2019; Choby and Skaar, 2016). Their characteristic tetrapyrrole macrocycle is comprised of four pyrrole rings arranged into a larger ring-like structure, as shown in **Figure 1** (Battersby et al., 1980). The center of this macrocycle often facilitates the binding of metal ions such as iron, as found in heme, cobalt in cobalamin, and magnesium in chlorophyll (Battersby et al., 1980). The chemical and physical properties of each porphyrin are shaped by its chelated metallic ion, as well as the modification of peripheral side chains (Battersby et al., 1980). Broadly, these structural features allow porphyrins to function either as light-absorbing chromophores or as facilitators of redox reactions.

These diverse roles have enabled porphyrin-centered processes to act as strategic nodes through which bacterial pathogenicity can be selectively modulated (Brzezowski et al., 2015; Choby and Skaar, 2016; Shimizu et al., 2019). Pathogenic bacteria frequently rely on elevated porphyrin production, remodeling, and acquisition to support virulence, stress tolerance, and persistence within hostile host environments, particularly under conditions imposed by host immunity and immune-mediated oxidative stress (Choby and Skaar, 2016; Butler et al., 2015; Barnard et al., 2020). In contrast, many commensal species limit porphyrin utilization due to the inherent cytotoxicity and pro-inflammatory consequences of these molecules, thereby constraining their dependence on porphyrin-associated pathways (Johnson et al., 2016; Byrd et al., 2018). This asymmetry creates a therapeutic window in which porphyrin-associated pathways can be exploited to impair pathogenic fitness while minimizing disruption to beneficial microbiota. Disrupting these pathways offers the opportunity to simultaneously attenuate multiple virulence-associated traits, including immune evasion, biofilm formation, and community-level cooperation, without necessarily eliminating bacterial viability (Heras et al., 2015; Barnard et al., 2020). This makes porphyrin-centered biology particularly well-suited to virulence-directed therapeutic strategies to steer bacterial evolution toward non-pathogenic or commensal-compatible states to combat AMR (Heras et al., 2015; Prestinaci et al., 2015).

In this review, we synthesize current understanding of porphyrin-centered processes in bacterial physiology and pathogenicity, emphasizing how these systems are co-opted during infection to enhance fitness and virulence (Choby and Skaar, 2016; Rowley and Kendall, 2019). We examine the roles of porphyrins in stress tolerance, metabolic adaptation, and polymicrobial community dynamics (Butler et al., 2015; Wollenberg et al., 2014; Barnard et al., 2020), and evaluate therapeutic strategies that target porphyrin-associated pathways with a focus on anti-virulence design. Finally, we discuss how advances in artificial intelligence (AI) and machine learning (ML) are reshaping target identification and lead discovery across porphyrin- and heme-dependent systems (Lluka and Stokes, 2023; Abduljalil et al., 2026). Together, these perspectives position porphyrin-centered biology as a promising frontier for next-generation antimicrobials.

## 2 Porphyrin-centered physiology enabling pathogenic potential in bacteria

### 2.1 Core porphyrin-dependent metabolic and redox capabilities

Porphyrins underpin a wide range of core metabolic and redox processes in bacteria through their incorporation into diverse cofactors and protein complexes. The conserved tetrapyrrole scaffold, combined with the capacity to chelate different metal ions, allows porphyrins to function as adaptable biochemical modules that integrate energy metabolism, electron transfer, redox homeostasis, and environmental sensing across highly variable growth conditions (Tanaka and Tanaka, 2007; Choby and Skaar, 2016; Shimizu et al., 2019).

Among porphyrin derivatives, heme is the most ubiquitous and functionally diverse. Iron-chelated protoporphyrin IX (PPIX), or heme as is more commonly known, is incorporated into hemoproteins that support cellular respiration, oxygen utilization, redox-driven enzymatic reactions, and gas-sensing pathways (Shimizu et al., 2019; Choby and Skaar, 2016). Through these functions, heme enables bacteria to sustain energy production, manage reactive oxygen species (ROS), and adapt respiration to fluctuating oxygen availability. Importantly, many heme-dependent proteins also participate in regulatory and sensory systems, allowing cells to couple metabolic state to environmental signals without requiring irreversible commitment to a single physiological mode (Shimizu et al., 2019).

Beyond heme, other porphyrin-derived cofactors similarly expand bacterial metabolic capacity. Cobalt-containing cobalamin (vitamin B12), for example, is essential for a range of enzymatic transformations involved in amino acid metabolism, odd-chain fatty acid processing, and carbon skeleton rearrangement (Rowley and Kendall, 2019). Because *de novo* cobalamin synthesis is restricted to a limited subset of bacterial and archaeal lineages, its availability introduces conditional metabolic flexibility and interspecies dependencies within microbial communities (Rowley and Kendall, 2019). In several host-associated bacteria, altered cobalamin availability has been shown to modulate transcriptional programs and metabolic outputs, highlighting the capacity of porphyrin-dependent cofactors to act as regulatory inputs as well as catalytic components (Kang et al., 2015; Forgie et al., 2023).

Collectively, porphyrin-dependent cofactors provide bacteria with a modular metabolic framework capable of supporting diverse physiological states rather than a single fixed lifestyle. By enabling the coordination of respiration, redox balance, metal utilization, and environmental sensing, these systems establish the biochemical capacity for adaptation under nutrient limitation, oxidative stress, and variable oxygen tension. While such capabilities are not intrinsically pathogenic, they form the physiological foundation upon which bacteria can later deploy specialized functions during host association and infection, as explored in subsequent sections.

### 2.2 Porphyrin biosynthesis and salvage as flexible metabolic mechanisms

**Figure 2:**
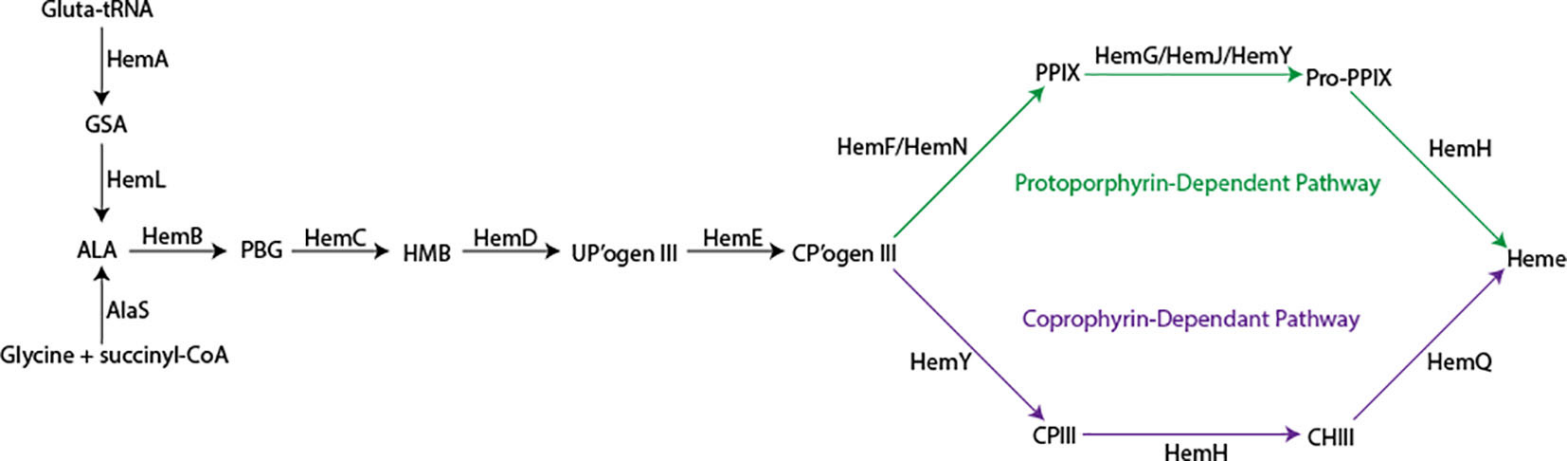
Bacterial heme biosynthesis pathways. The pathway initiates from either the glutamyl-tRNA route or the glycine-succinyl-CoA pathway, both converging on the universal precursor ALA, 5-aminolevulinic acid. Downstream intermediates proceed through PBG, porphobilinogen; HMB, hydroxymethylbilane; UP’ogen III, uroporphyrinogen III; and CP’ogen III, coproporphyrinogen III. From CP’ogen III, the pathway diverges into the PPIX-dependent branch (green) and the coproporphyrin-dependent branch (purple), which reconverge at the final product, heme. Abbreviations: gluta-tRNA, glutamyl-tRNA; succinyl-CoA, succinyl-coenzymeA; GSA, glutamate-1-semialdehyde; ALA, 5-aminolevulinic acid; PBG, porphobilinogen; HMB, hydroxymethylbilan; UP’ogen III, uroporphyrinogen III; CP’ogen III, coproporphyrinogen III; PPIX, protoporphyrin IX; pro-PPIX, protoporphyrinogen IX; CPIII, coproporphyrin III; CHIII, coproporphyrinogen III.

Porphyrin acquisition in bacteria follows two alternative strategies: *de novo* heme biosynthesis, which confers metabolic self-sufficiency but carries a substantial energetic cost, and heme scavenging, which exploits host-derived porphyrins and porphyrin metabolites to reduce biosynthetic burden during infections. Rather than relying on a single fixed pathway, bacteria exhibit considerable diversity in heme biosynthetic capacity, reflecting both phylogenetic history and adaptation to specific ecological and host-associated niches. Gram-positive bacteria predominantly utilize the coproporphyrin-dependent (CPD) pathway, which pivots around coproporphyrin III (CPIII), whereas Gram-negative bacteria more commonly rely on the protoporphyrin-dependent (PPD) pathway, which pivots around PPIX (**Figure 2**) (Dailey et al., 2015; Dali et al., 2023).

Regulation of porphyrin biosynthesis provides an additional layer of metabolic flexibility. In many bacteria, glutamyl-tRNA reductase (HemA) functions as a key regulatory checkpoint that senses intracellular heme abundance and modulates flux through the biosynthetic pathway accordingly (Jahn et al., 1991; Javor and Febre, 1992; Wang et al., 1997). Under heme-replete conditions, HemA activity and expression are suppressed, biasing cells toward utilization of exogenous porphyrins. Transcriptional regulators such as HrrA and ChrA in *Corynebacterium diphtheriae* (*C. diphtheriae)* further reinforce this heme-responsive feedback architecture (Bibb et al., 2007).

Oxygen availability also modulates porphyrin biosynthesis and regulation, allowing coordination between cofactor production, respiratory demand, and redox balance. In organisms such as *Staphylococcus aureus* (*S. aureus)* and *Bacillus subtilis*, oxygen-responsive regulatory networks adjust the expression of biosynthetic enzymes to sustain porphyrin-dependent metabolism across aerobic, microaerophilic, and anaerobic conditions (Mancini and Imlay, 2015; Choby and Skaar, 2016). Through the integration of biosynthesis, salvage, and regulatory feedback, bacteria maintain porphyrin supply without committing to a fixed metabolic strategy. Readers seeking a comprehensive mechanistic overview of these oxygen-dependent regulatory pathways are referred to the detailed excellent review by Choby and Skaar (2016).

Together, these mechanisms constitute a flexible porphyrin supply architecture that supports metabolic adaptability across diverse environmental contexts. While these capacities do not intrinsically confer pathogenicity, they establish the physiological readiness required for porphyrin-dependent processes to be differentially deployed under host-associated conditions, as discussed in subsequent sections.

### 2.3 Porphyrin acquisition systems as porphyrin and metal resource hubs

Porphyrin acquisition systems function as resource hubs that integrate extracellular capture, ligand discrimination, intracellular trafficking, and regulatory feedback to govern porphyrin-associated metal availability. Because free porphyrins are scarce in biological environments, bacteria have evolved specialized acquisition architectures capable of extracting heme and related cofactors from complex extracellular pools, including host-derived proteins and microbial sources (Choby and Skaar, 2016). Importantly, these systems are not limited to passive import; rather, they coordinate multiple molecular interactions to control the timing, specificity, and routing of porphyrins into downstream metabolic pathways.

Across bacterial taxa, porphyrin acquisition systems vary substantially in architecture and substrate preference, reflecting adaptation to distinct ecological niches. Despite this diversity, they converge functionally on the same core role: linking extracellular porphyrin availability to intracellular metabolic state. As such, porphyrin acquisition systems occupy a central position at the interface between environmental resource landscapes and intracellular regulatory networks.

#### 2.3.1 Gram-negative porphyrin acquisition networks

**Figure 3:**
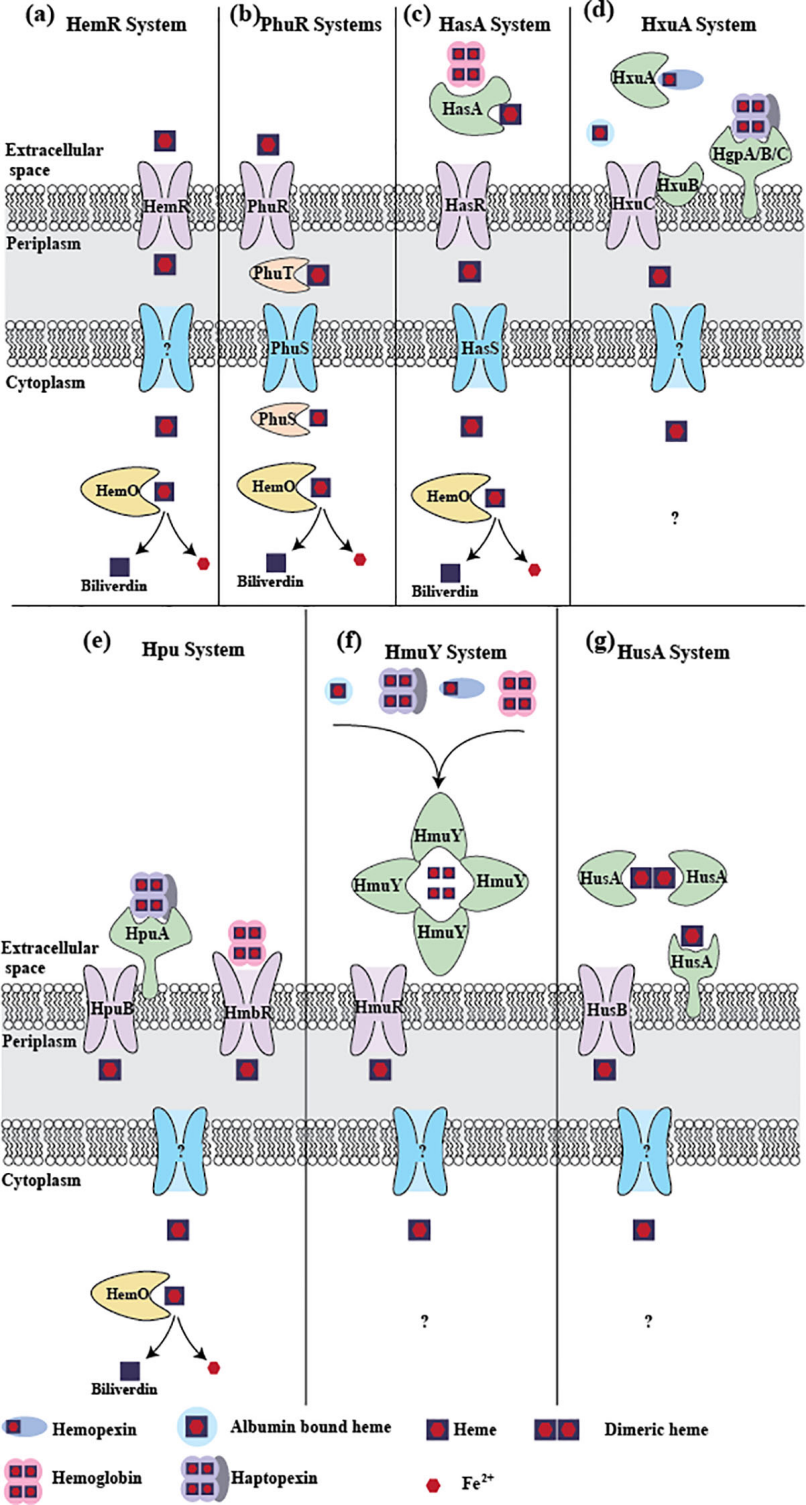
Gram-negative heme uptake systems. **(A)** The HemR and **(B)** PhuR systems represent non-hemophore-based heme uptake pathways in which outer-membrane receptors HemR and PhuR directly acquire heme. Heme is transported across the inner membrane by an unidentified transporter in the HemR system and by PhuUV in the PhuR system, after which it is degraded into biliverdin and Fe^2+^, free iron by HemO. The Phu system additionally employs the cytosolic chaperones PhuT, which shuttles heme between transport components, and PhuS, which stores heme and delivers it to HemO. **(C)** The HasA system functions similarly to HemR but incorporates the secreted hemophore HasA, which binds heme or hemoglobin and transfers heme to the outer-membrane receptor HasR, then inner membrane transporter HasS, for internalization, followed by degradation into biliverdin and free iron by HemO. **(D)** The HxuA system uses a secreted hemophore-like protein to bind hemopexin and liberate heme for uptake by the outer-membrane receptor HxuC, assisted by HxuB. HxuC can also acquire heme from serum albumin. Additional membrane-bound proteins HgpA, HgpB, and HgpC bind heme from haptoglobin, hemoglobin, or myoglobin (only haptoglobin binding shown). **(E)** The Hpu system does not secrete a hemophore; instead, the surface-bound protein HpuA binds hemoglobin and haptoglobin, releasing heme for uptake by the associated outer-membrane receptor HpuB. The receptor HmbR can independently extract heme from hemoglobin. Internalized heme is transported across the inner membrane by an unidentified transporter and degraded into biliverdin and free iron by HemO. **(F)** In the HmuY system, the secreted hemophore HmuY extracts heme from hemopexin, albumin, hemoglobin, or haptoglobin as a tetramer and transfers it to the outer-membrane receptor HmuR for internalization. **(G)** The HusA system expresses HusA as both a membrane-bound heme-binding protein and a secreted hemophore that preferentially binds dimeric heme as a dimer. Figure adapted from ‘Metallotherapeutics Development in the age of iron-clad bacteria’ (Centola et al., 2020) and ‘Heme Synthesis and Acquisition in Bacterial Pathogens’ (Choby and Skaar, 2016).

In Gram-negative bacteria, high-affinity porphyrin acquisition is primarily mediated by TonB-dependent receptors. Receptors such as HemR in *Serratia marcescens* (*S. marcescens*), and PhuR in *Pseudomonas aeruginosa* (*P. aeruginosa*) mediate direct heme uptake when environmental concentrations are high (Benevides-Matos and Biville, 2010; Otero-Asman et al., 2019). However, because free heme is typically scarce during infection, many pathogens rely on hemophore-based systems to expand their access. Secreted or surface-exposed hemophores, including HasA in *S. marcescens* and *P. aeruginosa*, HusA and HmuY in *Porphyromonas gingivalis (P. gingivalis)*, and HpuA in *Neisseriaceae* species, capture free heme or actively extract it from hemoproteins (**Figure 3**) (Gao et al., 2018; Wojtowicz et al., 2009; Wong et al., 2015; Noinaj et al., 2012; Choby and Skaar, 2016; Sheldon et al., 2016; Gao et al., 2010). These hemophores subsequently deliver their cargo to cognate TonB-dependent transporters, ensuring efficient uptake even under intense nutritional immunity.

Several Gram-negative species deploy layered and partially redundant acquisition strategies. The HxuCBA pathway in *P. aeruginosa* and *Haemophilus influenzae*, further broaden heme acquisition capacity through multiple, partially redundant receptors capable of binding hemopexin, haptoglobin, hemoglobin, myoglobin, or albumin-bound heme (Otero-Asman et al., 2019; Sheldon et al., 2016). The HpuA system similarly incorporates the hemoglobin-binding transporter HmbR to extract and internalize heme (Choby and Skaar, 2016). Collectively, the coexistence of these layered strategies illustrates how Gram-negative pathogens assemble robust, flexible uptake networks that allow them to handle fluctuating host microenvironments (**Figure 3**).

#### 2.3.2 Gram-positive porphyrin acquisition strategies

**Figure 4:**
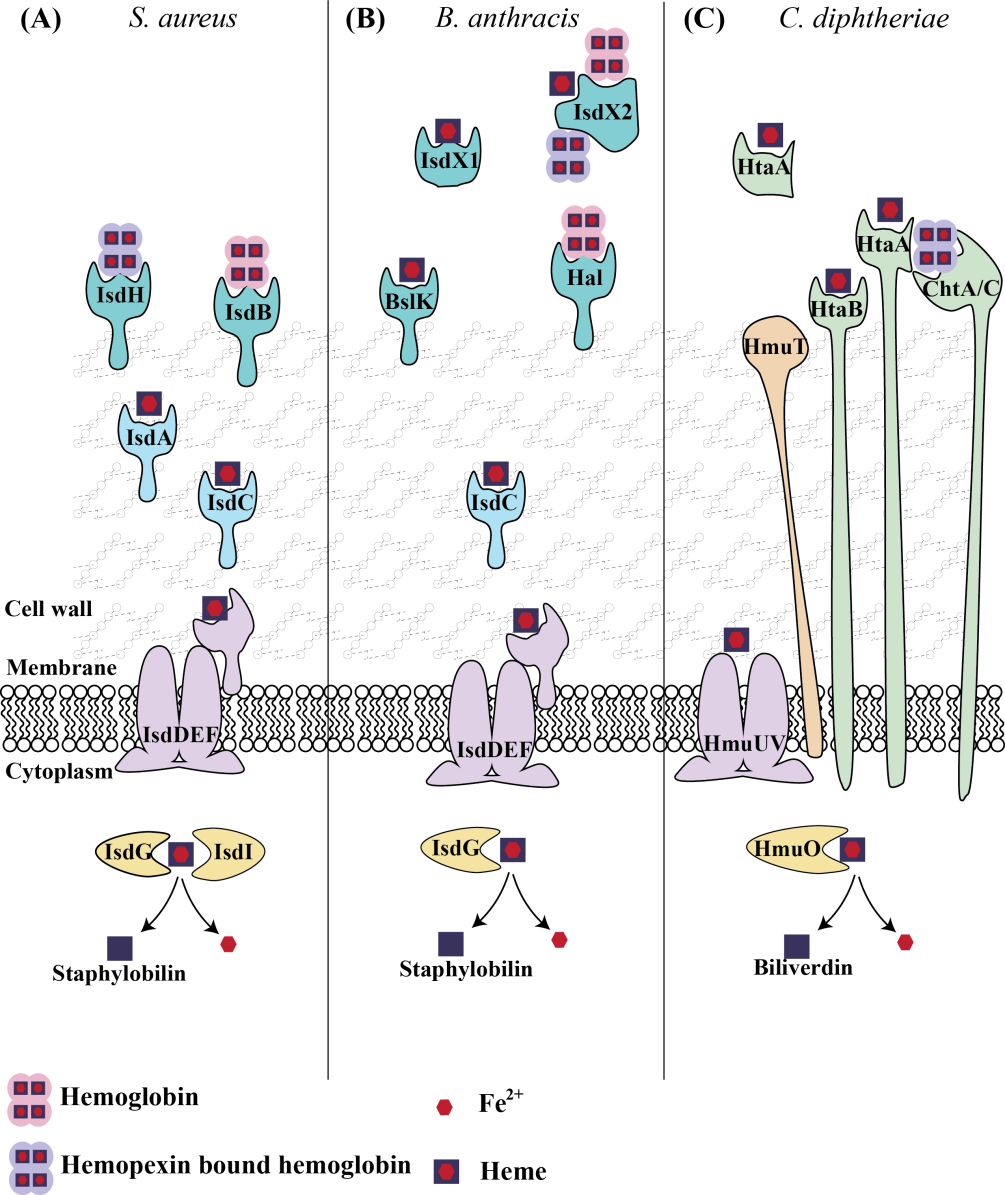
Gram-positive heme uptake systems. **(A)** *Staphylococcus aureus*: the Isd, iron-regulated surface determinant system mediates heme acquisition. Cell wall-anchored IsdH is the primary receptor for hemopexin-bound hemoglobin, while IsdB serves as the main hemoglobin receptor. IsdA binds free heme or accepts heme from IsdB or IsdH and transfers it to IsdC, which subsequently passes heme to the IsdDEF transporter for internalization. Internalized heme is degraded into staphylobilin and Fe^2+^, free iron by the heme oxygenases IsdG and IsdI. **(B)** *Bacillus anthracis*: the Isd system variant includes the cell wall-bound receptor Hal, which binds hemoglobin, and the secreted hemoprotein IsdX2, which can bind hemoglobin, hemopexin, and free heme. Cell wall-anchored BslK and the secreted protein IsdX1 also bind free heme. Heme is transferred through the cell wall via IsdC to the IsdDEF transporter for internalization and is subsequently degraded into staphylobilin and free iron by the heme oxygenase IsdG. **(C)** *Corynebacterium diphtheriae*: heme acquisition is mediated by the cell-wall-spanning lipoprotein HtaA, which binds free heme or, together with ChtA or ChtC, acquires heme from hemopexin. A secreted form of HtaA also binds free heme. HtaB binds free heme or accepts heme from HtaA and transfers it to the HmuTUV transporter for internalization. Internalized heme is degraded into biliverdin and free iron by the heme oxygenase HmuO. Figure adapted from ‘Heme synthesis and acquisition in bacterial pathogens’ (Choby and Skaar, 2016).

Gram-positive bacteria employ distinct but functionally analogous systems. The iron-regulated surface determinant (Isd) pathway in *S. aureus* and *Bacillus anthracis* (*B. anthracis)* exemplifies this architecture. Near Iron Transporter (NEAT) domain proteins such as IsdA, IsdB, IsdH, IsdX1/X2, Hal, and BslK bind hemoglobin or hemopexin, extract heme, and relay it across the cell wall to the membrane transporter IsdDEF (Choby and Skaar, 2016; Sheldon et al., 2016). Other Gram-positive species, including *C. diphtheriae*, rely on non-NEAT systems such as HmuTUV, HtaABC, and ChtA/C, which similarly coordinate heme extraction and channeling to the ATP-binding cassette (ABC) transporter HmuUV (**Figure 4**) (Choby and Skaar, 2016). Despite structural differences, these pathways converge on the same virulence-enhancing outcome: reliable access to porphyrins and iron within host niches where these resources are otherwise stringently restricted.

#### 2.3.3 Intracellular processing and storage as a regulatory control center

Following acquisition, intracellular porphyrin processing and storage function as a regulatory control center that governs iron release, sequestration, and redox balance in response to metabolic demand and environmental stress. Once internalized, heme is either incorporated directly into hemoproteins or degraded to release iron and porphyrin metabolites for downstream processes (Choby and Skaar, 2016). Bacterial heme oxygenases such as HemO in Gram-negative *Neisseria* species and HmuO in Gram-positive *C. diphtheriae* cleave heme into biliverdin and free iron, occasionally with the assistance of chaperones like PhuS in *P. aeruginosa* (Choby and Skaar, 2016; Mourino et al., 2016). Alternative oxygenases, including IsdG in *B. anthracis* and the IsdG/IsdI homodimers in *S. aureus*, generate staphylobilin and iron (Skaar et al., 2006; Choby and Skaar, 2016). Importantly, heme degradation is tightly coupled to mechanisms that prevent iron-mediated toxicity. Excess free iron is sequestered by storage proteins such as bacterioferritin B (BfrB) in *P. aeruginosa*, preventing oxidative damage from iron-catalyzed ROS while allowing pathogens to retain access to iron reserves (Eshelman et al., 2017).

Together, the metabolic capabilities, biosynthetic and salvage pathways, acquisition systems, and intracellular control mechanisms described above establish the physiological capacity for porphyrin utilization in bacteria. How these capacities are functionally deployed during host association and infection to drive virulence, stress tolerance, and community-level behaviors is addressed in the following section.

## 3 Porphyrins as mediators of virulence in bacterial pathogens

### 3.1 Porphyrin production, utilization and pathogenicity

Elevated bacterial porphyrin utilization, particularly through porphyrin acquisition and scavenging systems, is strongly associated with increased pathogenicity and virulence. Because hosts tightly regulate and sequester porphyrins as part of nutritional immunity, pathogens often gain access to these molecules only when inflammation disrupts host tissues. This disruption may be triggered directly by porphyrin-scavenging pathogens or by other members of a polymicrobial community (Choby and Skaar, 2016). As a result, many pathogenic species have evolved extensive genetic repertoires dedicated to porphyrin acquisition, modification, and incorporation, including systems capable of exploiting bacterially altered host-derived porphyrins (Choby and Skaar, 2016). These metabolic capabilities contribute to multiple virulence-associated traits: elevated porphyrin levels promote aggregation, biofilm formation, and heme synthesis, and can intensify inflammatory responses in human cells (Barnard et al., 2020). Consistent with this, porphyrin production is disproportionately associated with pathogenic bacteria and one study reported that 28 of 32 wound-infecting isolates produced porphyrins (Jones et al., 2020).

Although the metabolic flexibility afforded by porphyrin modification appears broadly advantageous, commensal bacteria often exhibit constraints in their use of these molecules due to their pro-inflammatory properties. Pathogenic and opportunistic strains, by contrast, frequently produce substantially higher porphyrin levels than their commensal counterparts. This pattern is well illustrated in *Cutibacterium acnes* (*C. acnes*, formerly *Propionibacterium acnes*), where opportunistic strains generate markedly more porphyrins, particularly under conditions that favor virulence, such as altered nutrient availability or increased disease severity (Kang et al., 2015; Johnson et al., 2016; Byrd et al., 2018). Commensal strains do not show comparable increases in porphyrin synthesis or associated pathogenic traits under similar pressures (Johnson et al., 2016). These elevated porphyrin levels have direct consequences for host inflammation. Porphyrin-induced K^+^ leakage in keratinocytes infected with acne-associated *C. acnes* strains, or exposed to their extracted porphyrins, activates the inflammasome and drives inflammatory cytokine release (Spittaels et al., 2021). Thus, porphyrin overproduction not only marks pathogenic strains but actively contributes to their inflammatory potential.

Porphyrin production can also shift dynamically in response to microbial interactions within polymicrobial communities, further reinforcing its role in pathogenicity. Fluorescence-based studies show that opportunistic species such as *Fusobacterium nucleatum (F. nucleatum)* increase production of virulence-associated porphyrins when co-cultured with keystone pathogens like *P. gingivalis* (Lee et al., 2018). Transcriptional analyses support this observation; genes involved in porphyrin metabolism, including *cobK* and *cbiJ*, are upregulated in *F. nucleatum* during co-culture with *P. gingivalis* (Yamaguchi-Kuroda et al., 2023). This shift is attributed to the sudden availability of porphyrin metabolites shared by the pathogenic colonizing bacteria (Lee et al., 2018). Such cooperative metabolic interactions amplify virulence potential within polymicrobial infections, enabling bacteria to collectively enhance porphyrin availability and exploit it for pathogenic gain.

Taken together, these findings underscore a consistent theme: elevated or altered porphyrin production is not merely a metabolic byproduct but a hallmark and driver of bacterial pathogenicity. Whether through host-induced inflammation, strain-specific metabolic capacity, or polymicrobial cooperation, enhanced porphyrin utilization provides pathogens with a potent means of promoting inflammation, persistence, and tissue damage.

### 3.2 Porphyrins as coping mechanisms of environmental stress

The structural plasticity of porphyrins and their derivatives provides bacteria with versatile mechanisms to withstand environmental stress, many of which directly enhance virulence and pathogenicity. A prominent example is the use of modified porphyrins to mitigate oxidative stress. Iron (Fe^2+^) is incorporated by most bacteria into a wide range of essential complexes and pathways, including iron-sulfur-dependent heme required for cellular respiration and numerous hemoprotein-mediated processes. However, iron is readily oxidized and interacts with molecular oxygen in aerobic environments, generating elevated ROS levels (Butler et al., 2015; Choby and Skaar, 2016). This oxidative burden becomes particularly significant during host immune responses, such as the oxidative burst initiated by host innate immune systems (Capek and Vecerek, 2023).

Manganese (Mn^3+^), while functionally similar to iron in many biochemical contexts, exhibits substantially greater resistance to oxidation (Butler et al., 2015; Choby and Skaar, 2016). Several pathogens exploit this difference by substituting manganese for iron within porphyrin scaffolds. One well-characterized example is the incorporation of manganese-PPIX (MnPPIX) in place of iron-containing heme (FePPIX) by *P. gingivalis* (Butler et al., 2015). This environmentally responsive exchange reduces oxidative stress within the cell, enabling survival in highly oxidative niches and diminishes the effectiveness of host oxidation-based immune defenses. By buffering against ROS-mediated damage, MnPPIX incorporation supports persistence within inflamed tissues and facilitates deeper infiltration into host environments (Capek and Vecerek, 2023).

Such adaptive modifications illustrate how porphyrin plasticity contributes not only to stress tolerance but also to enhanced virulence. By enabling pathogens to withstand oxidative immune pressures, porphyrin-based coping mechanisms play a central role in shaping the outcome of host-pathogen interactions and represent an important consideration in the development of therapeutics aimed at disrupting bacterial survival strategies.

### 3.3 Porphyrin-driven community-wide virulence

Porphyrin use by pathogenic and opportunistic bacteria can amplify virulence across entire polymicrobial communities. This community-level influence arises in part from shared regulatory architectures that prioritize the scavenging of exogenous porphyrins over endogenous synthesis. Heme-dependent repression of *hemA* exemplifies this conserved hierarchy (Wang et al., 1997; Jahn et al., 1991; Javor and Febre, 1992; Bibb et al., 2007). When porphyrin metabolites suddenly become available, such as when released by keystone pathogens like *P. gingivalis*, neighboring opportunists, including *F. nucleatum*, rapidly shift toward exogenous porphyrin uptake (Lee et al., 2018). These coordinated metabolic adjustments initiate a positive feedback loop: destructive, inflammation-inducing porphyrin acquisition systems liberate additional host-sequestered porphyrins, enriching the local environment and further encouraging surrounding bacteria to favor scavenging over biosynthesis (Choby and Skaar, 2016). In this way, porphyrin availability becomes a shared resource that reshapes community behavior and collectively enhances virulence.

Porphyrin acquisition systems also intensify these dynamics by enabling pathogens to monopolize or redirect shared nutritional resources. The high-affinity hemophore HmuY of *P. gingivalis*, for example, can strip heme from homologous proteins in *Bacteroides fragilis*, effectively converting co-infecting species into auxiliary heme reservoirs (Antonyuk et al., 2023). This competitive exploitation strengthens *P. gingivalis*’s position within the community while simultaneously altering the nutritional landscape for other microbes. Such resource reallocation aligns with broader observations linking elevated porphyrin levels to increased aggregation, biofilm formation, and heightened inflammatory responses in human cells (Barnard et al., 2020). Thus, porphyrin-mediated interactions not only enhance the fitness of individual pathogens but also restructure the microenvironment in ways that promote collective virulence.

Beyond shaping microbial competition, porphyrins can directly modulate host immune responses, further influencing community-wide pathogenicity. In dual-species biofilms formed by *Fusobacterium necrophorum* and *Porphyromonas levii*, accumulation of PPIX suppresses neutrophil ROS production and chemotaxis, weakening early innate immune defenses and enabling biofilm persistence (Lockhart et al., 2022). Although similar immunosuppressive effects occur in planktonic cultures, they are markedly amplified within porphyrin-rich biofilms, highlighting the importance of spatially concentrated porphyrin pools in shaping infection outcomes.

Porphyrins also function as interspecies signaling molecules that promote dysbiosis and cooperative virulence. As noted earlier, opportunistic strains of *C. acnes* increase porphyrin production under conditions favoring pathogenicity, but this shift also triggers downstream effects on coinfecting bacteria (Johnson et al., 2016). Extracellular CPIII produced by *C. acnes* induces aggregation and biofilm attachment in *S. aureus*, strengthening mixed-species communities and enhancing their resilience (Wollenberg et al., 2014). These signaling-driven interactions illustrate how porphyrin metabolites can coordinate community behavior, promoting structural stability and collective persistence.

Recent evidence further supports the concept that porphyrin remodeling can act as a community-level virulence driver rather than a purely intracellular adaptation. FetB-dependent production of MnPPIX by *P. gingivalis* has been shown to actively reshape the surrounding microbial community, promoting dysbiosis and altering the metabolic balance of the oral microbiota (Phonok et al., 2026). In this context, MnPPIX functions not only as a stress-protective porphyrin within the bacteria but also as a surface metabolic signal that influences neighboring species. This finding reinforces the idea that porphyrin remodeling can position keystone pathogens as architects of dysbiotic niches, amplifying virulence through ecological rather than purely cell-autonomous mechanisms.

Together, these observations demonstrate that porphyrin dynamics extend far beyond individual pathogens. By reshaping nutrient availability, modulating immune responses, and acting as interspecies cues, porphyrins orchestrate community-wide shifts that enhance cooperative virulence and infection persistence. These insights position porphyrin-modulating strategies as promising avenues for disrupting polymicrobial pathogenic networks.

## 4 Enzymatic and chemical modification of porphyrins and virulence

### 4.1 Porphyrin chelatases

**Figure 5:**
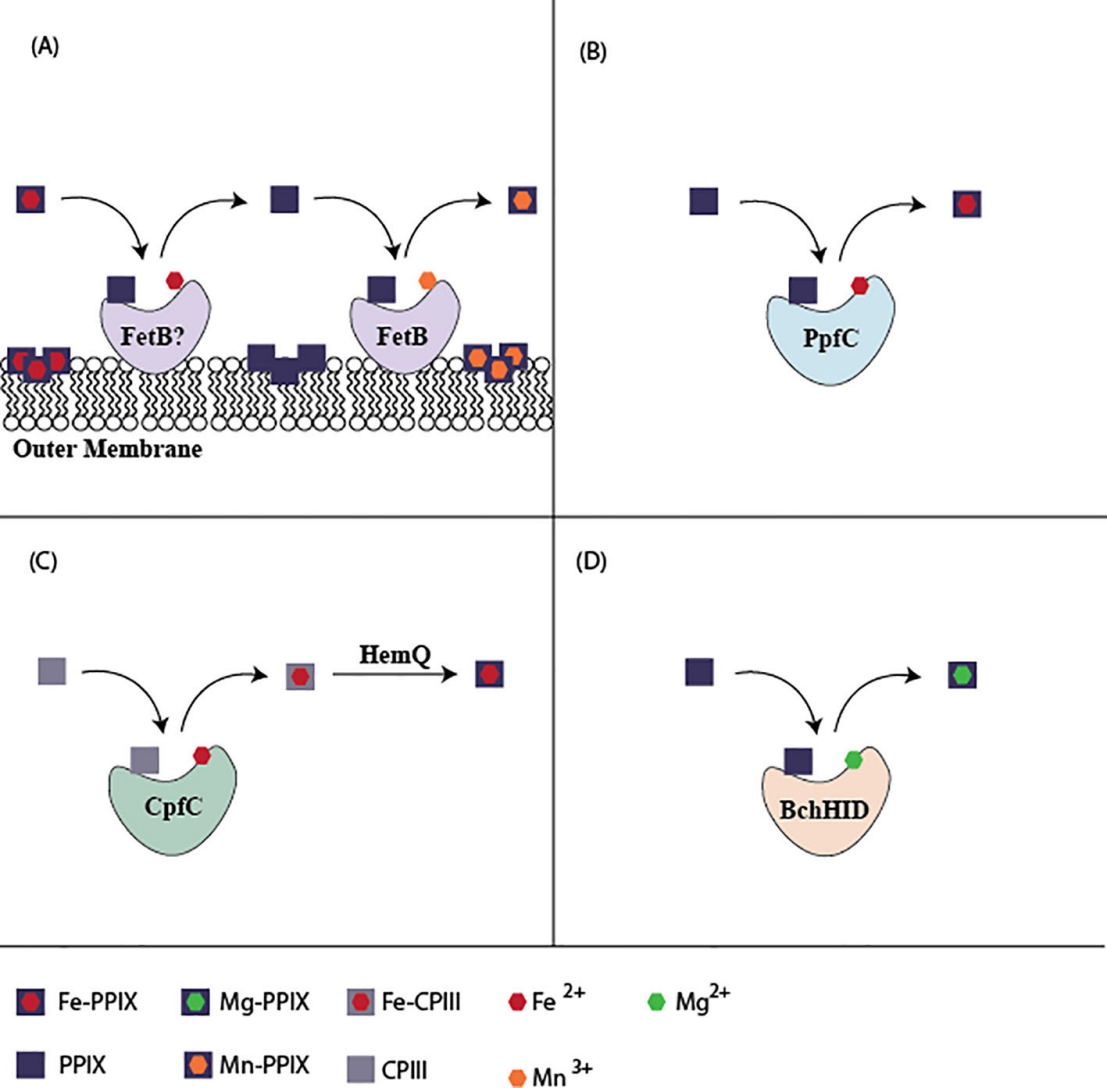
Porphyrin chelatase-mediated synthesis of metallic porphyrins. A schematic overview of the enzymatic pathways responsible for generating distinct metalloporphyrins. **(A)** In *Porphyromonas gingivalis*, FePPIX, heme is first dissociated into PPIX, protoporphyrin IX and Fe^2+^, free iron by an unidentified enzyme, potentially FetB, indicated as 'FetB?' in the figure. FetB subsequently inserts manganese into PPIX, producing MnPPIX, manganese-PPIX. The porphyrins accumulate on the surface of the outer membrane when not being actively processed or internalised for use. **(B)** In the PPD, protoporphyrin-dependent pathway; PpfC, protoporphyrin IX ferrochelatase catalyses the chelation of PPIX and iron, producing FePPIX. **(C)** In the CPD, coproporphyrin-dependent pathway; CpfC, coproporphyrin III ferrochelatase catalyses the CPIII, chelation of coproporphyrin III and Fe-CPIII, iron, producing coproporphyrinogen III, which is then converted to FePPIX by HemQ. **(D)** In *Rhodobacter sphaeroides* a manganese-chelatase, BchHID, catalyses the chelation of PPIX and magnesium into MgPPIX, magnesium PPIX.

The remarkable functional versatility of porphyrins is underpinned by a diverse suite of enzymes, known as porphyrin chelatases, capable of chemically modifying their metal centers. While chelators in general are molecules that bind metal ions, porphyrin chelatases specifically catalyze the insertion, removal, or substitution of metals within the porphyrin macrocycle. This capacity allows bacteria to tailor porphyrin properties to shifting environmental pressures, including those imposed by host immunity, such as oxidative stress or metal sequestration. These enzymatic modifications directly support pathogenic survival. By altering the metal content of porphyrins, pathogens can optimize redox behavior, evade host defenses, and maintain metabolic activity in hostile niches. For example, *P. gingivalis* does not rely on acquiring endogenous MnPPIX. Instead, it actively exchanges the metal ions within porphyrins using the chelatase FetB, enabling rapid adaptation to fluctuating oxidative conditions during infection (**Figure 5**) (Butler et al., 2015; Phonok et al., 2026). Such flexibility enhances resilience in inflamed, immune-mediated oxidative attacks and contributes to the organism’s virulence.

Beyond their adaptive value, porphyrin chelatases are embedded within essential biosynthetic pathways. Ferrochelatases, for instance, are indispensable enzymes in both the CPD and PPD heme biosynthesis pathways. In the CPD pathway, coproporphyrin III ferrochelatase (CpfC) inserts iron into CPIII to form coproheme, which is subsequently decarboxylated to heme. In the PPD pathway, protoporphyrin IX ferrochelatase (PpfC) directly inserts iron into PPIX to generate heme (**Figure 5**) (Dailey et al., 2017). Because heme is essential for respiration, redox balance, and numerous virulence-associated enzymes, these chelatases indirectly support pathogenic fitness. Porphyrin chelatases also extend beyond heme biosynthesis into specialized metabolic systems. Photosynthetic bacteria such as *Rhodobacter sphaeroides* (*R. sphaeroides*) employ a manganese chelatase complex (BchHID) to generate bacteriochlorophyll (BChl), a pigment central to their photosynthetic machinery (**Figure 5**) (Jaschke et al., 2011). Although not directly linked to virulence, this example highlights the evolutionary breadth of porphyrin chelatases and their capacity to diversify porphyrin function across biological contexts.

Together, these examples illustrate the wide functional range of porphyrin chelatases, from essential metabolic enzymes to adaptive tools that enhance survival in immune-restricted environments. Their ability to remodel porphyrins in response to host-imposed pressures underscores their relevance to pathogenicity and positions them as potential targets for therapeutic intervention.

### 4.2 Porphyrin remodeling and niche specialization

Porphyrin chelatases play a central role in diversifying the metalation states of porphyrins, enabling bacteria to fine-tune these molecules to meet the demands of specific ecological and host-associated niches. By altering the metal inserted into the porphyrin scaffold, bacteria can modulate redox properties, stability, and resistance to metal toxicity, traits that directly influence survival under immune pressure and other environmental stresses.

In the bacteriochlorophyll (BChl) synthesis pathway, magnesium (Mg^2+^) is typically inserted into intermediates such as magnesium PPIX (MgPPIX). However, *Acidiphilium rubrum* (*A. rubrum)* and a mutant strain of *R. sphaeroides* demonstrate an alternative strategy: the use of zinc chelatases capable of substituting zinc (Zn^2+^) for magnesium (Jaschke et al., 2011; Jaime-Perez et al., 2021). In *A. rubrum*, this zinc substitution provides a distinct advantage in metal-rich, acidic environments. Because zinc-bound porphyrins resist displacement by toxic metals such as copper (Cu^2+^), they prevent the formation of copper porphyrins that would otherwise disrupt photosynthetic function. This stability preserves cellular integrity under heavy metal stress and reflects a specialized adaptation to the extreme niches *A. rubrum* inhabits (Jaime-Perez et al., 2021). A similar adaptation is observed in the *R. sphaeroides* mutant lacking a functional magnesium chelatase. In this strain, a ferrochelatase instead generates zinc protoporphyrin IX (ZnPPIX), effectively replacing MgPPIX within the BChl pathway. Prior to this discovery, the ability of ferrochelatases to synthesize ZnPPIX for use in BChl biosynthesis had only been documented in *A. rubrum* (Jaschke et al., 2011). Together, these findings suggest that zinc substitution may represent an ancestral strategy for coping with metal toxicity, with zinc-bound porphyrins offering enhanced stability in environments where magnesium-porphyrins are vulnerable to displacement.

Porphyrin remodeling also plays a critical role in pathogenic niche specialization. The incorporation of manganese into porphyrins, rather than canonical iron, has been pivotal in the emergence of *P. gingivalis* as a keystone oral pathogen. *P. gingivalis’s* ability to synthesize MnPPIX, combined with its heme-auxotrophic lifestyle, is a major contributor to its pathogenicity (Tribble et al., 2013). The enhanced oxidative protection afforded by MnPPIX relative to iron-based heme, combined with the bacterium’s capacity to utilize host-derived heme in MnPPIX biosynthesis, enables *P. gingivalis* to exploit host resources while simultaneously enhancing its resilience to immune-mediated oxidative damage (Butler et al., 2015; Tribble et al., 2013). These examples highlight the evolutionary significance of porphyrin chelatases in shaping bacterial diversification, environmental specialization, and virulence. By enabling bacteria to remodel porphyrins in response to metal availability, immune pressure, and niche-specific constraints, chelatases serve as powerful drivers of adaptive potential and pathogenic success.

## 5 Porphyrin/heme pathways as therapeutic targets

Porphyrin- and heme-associated pathways have long been explored as antimicrobial targets due to their central roles in bacterial metabolism, redox homeostasis, and pathogenic fitness. Early therapeutic efforts provided important proof-of-principle that these systems are vulnerable to pharmacological intervention, but they also revealed recurring challenges, including host toxicity, limited specificity, and the remarkable adaptability of bacterial metal acquisition networks (Centola et al., 2020; Repac Antic et al., 2022). These limitations have increasingly motivated a shift away from broad survival-targeting approaches toward strategies that selectively disrupt porphyrin-dependent virulence functions and regulatory feedback while preserving commensal balance and minimizing selective pressure for AMR.

### 5.1 Metal deprivation and overload as survival-targeting strategies

#### 5.1.1 Metal chelators

Initial approaches to disrupting porphyrin-associated processes focused on depriving pathogens of essential metals using classical chelators. Agents such as Ethylenediaminetetraacetic acid (EDTA) and deferoxamine (DFO) demonstrated that iron sequestration could suppress bacterial growth, including pathogens such as *P. gingivalis*, but systemic application proved incompatible with host metal homeostasis (Moon et al., 2011; Repac Antic et al., 2022). Even later-generation chelators such as nitroxoline, which improved antibacterial activity and inhibited adhesion and biofilm formation, retained broad metal-binding profiles that produced off-target effects on host cells and commensal microbes (El Sakka and Gould, 2016; Wijma et al., 2018; Repac Antic et al., 2022). Collectively, these outcomes underscored a key limitation: global metal deprivation imposes strong selective pressure while offering limited scope for virulence-specific targeting.

#### 5.1.2 Metal transporter disruptors

A complementary survival-targeting strategy sought to induce toxic intracellular accumulation of metals by dysregulating bacterial transport systems. Small-molecule modulators capable of driving lethal accumulation of iron, copper, and zinc in Gram-positive bacteria demonstrated potent bactericidal effects (Juttukonda et al., 2020). However, interference with conserved host metal transport pathways led to unacceptable toxicity, and the broad-spectrum nature of these agents increased the risk of AMR. These early strategies collectively illustrated that direct perturbation of metal availability primarily targets bacterial survival rather than pathogenic behavior, limiting their suitability for long-term control of AMR.

### 5.2 Intracellular control of porphyrin-associated virulence

#### 5.2.1 Iron mobilization inhibitors

**Figure 6:**
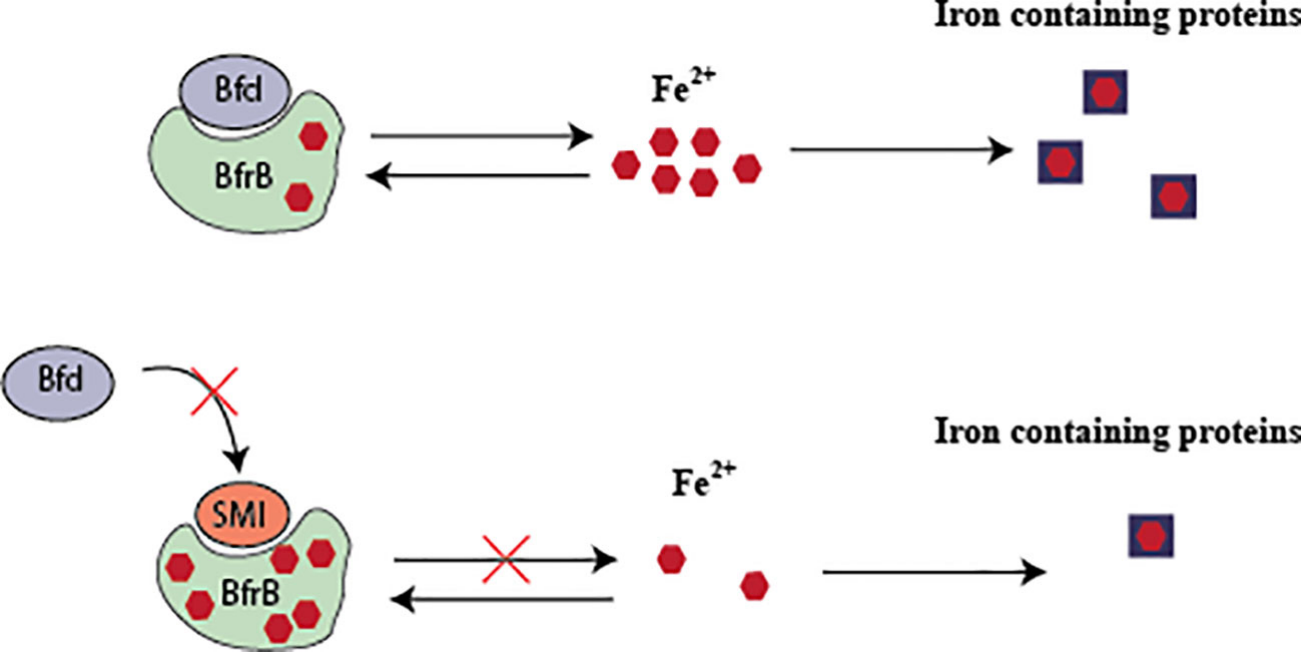
Inhibition of BfrB, bacterioferritin B and Bfd, bacterioferritin-associated ferredoxin interactions. In the *Pseudomonas aeruginosa* BfrB-Bfd system, excess intracellular Fe^2+^, iron is stored within BfrB. Mobilization occurs when the ferredoxin Bfd binds BfrB, enabling the release of iron for incorporation into iron-dependent proteins. Binding of a small-molecule inhibitor to BfrB prevents Bfd association, thereby blocking iron mobilization. This results in irreversible sequestration of iron within BfrB and depletion of accessible intracellular iron reserves.

More recent approaches have focused on intracellular porphyrin-associated processes that operate downstream of uptake, where compensatory acquisition strategies are less effective. Iron mobilization inhibitors targeting BfrB in *P. aeruginosa* exemplify this shift. By disrupting the BfrB– bacterioferritin-associated ferredoxin (Bfd) interaction, these inhibitors prevent the release of intracellular iron stores, impairing respiration, redox balance, and biofilm formation even under iron-replete conditions (**Figure 6**) (Punchi Hewage et al., 2019; Soldano et al., 2020; Eshelman et al., 2017). Importantly, iron-mobilization inhibitors also potentiate the activity of conventional antibiotics, positioning intracellular iron control as a virulence-linked vulnerability with therapeutic synergy potential (Punchi Hewage et al., 2019). By acting downstream of iron acquisition, this strategy limits the effectiveness of compensatory uptake pathways and positions intracellular iron mobilization as a virulence-linked control node rather than a generic bactericidal target.

#### 5.2.2 Inhibition of heme degradation

Heme degradation represents another intracellular control point with strong anti-virulence relevance. Blocking heme catabolism not only prevents iron liberation but also triggers negative feedback that reduces heme uptake and dampens heme-sensing capabilities (Dent et al., 2019; Mourino et al., 2016). This dual effect makes heme degradation an appealing target for anti-virulence strategies, as it disrupts iron acquisition while simultaneously impairing regulatory pathways that coordinate heme-dependent metabolism. Small-molecule inhibitors (SMIs) and allosteric iminoguanidine-based compounds targeting HemO reduce *P. aeruginosa* virulence *in vitro* while minimizing reliance on bactericidal activity (Hom et al., 2013; Liang et al., 2018; Heinzl et al., 2016). However, as with other intracellularly acting antimicrobial strategies, heme-degradation inhibitors are vulnerable to resistance mechanisms that limit drug penetration or promote efflux (Heinzl et al., 2016; Hom et al., 2013; Liang et al., 2018). Although challenges related to cellular permeability and efflux remain, these studies demonstrate how interfering with porphyrin handling rather than porphyrin availability can destabilize pathogenic fitness with reduced selective pressure.

### 5.3 Disruption of porphyrin acquisition: mimicry and transcriptional control

Porphyrin and metal mimics represent a distinct therapeutic logic that exploits bacterial acquisition systems instead of blocking them. Gallium (Ga³^+^), which closely resembles iron but lacks redox activity, enters cells through iron- and heme-uptake pathways and disrupts redox-dependent processes essential for growth and virulence (Centola et al., 2020). Clinical evaluation of gallium nitrate (Ganite) demonstrated efficacy against *P. aeruginosa* in cystic fibrosis patients with minimal observed resistance (Goss et al., 2018).

Incorporating gallium into porphyrin mimics such as Gallium PPIX (GaPPIX) and Gallium mesoporphyrin IX (GaMPIX) further enhanced uptake through heme-specific transport systems and improved activity against biofilms and multidrug-resistance (MDR) pathogens (Chang et al., 2016; Arivett et al., 2015; Hijazi et al., 2017). These compounds outperform Ganite in several contexts, underscoring the value of targeting heme-specific uptake systems and demonstrate that pathway-specific mimicry can achieve selectivity and may lead to a lower propensity for resistance than conventional bactericidal agents.

In addition to direct pathway hijacking through structural mimicry, porphyrin acquisition can also be disrupted indirectly through modulation of virulence-associated transcriptional programs rather than direct blockade of uptake machinery. Suppressing exogenous heme acquisition can activate regulatory feedback loops that downregulate heme uptake genes while increasing reliance on endogenous porphyrin biosynthesis (Section 2.2), a shift that is often associated with reduced virulence. Externally exposed heme receptors, including the *S. aureus* hemoglobin-binding proteins IsdH and IsdB, represent particularly attractive nodes for such modulation due to their surface accessibility.

Consistent with this concept, the flavonoid 2R,3R-dihydromyricetin (DMY) has been shown to exert anti-virulence effects in *S. aureus* by suppressing expression of key Isd system components, including IsdA, IsdB, and IsdC, while destabilizing metabolic homeostasis and biofilm formation (Ran et al., 2023b). Although DMY also displays bactericidal activity, its ability to transcriptionally downregulate heme acquisition systems highlights a broader therapeutic strategy in which pathogens are shifted away from dependence on exogenous heme scavenging toward less virulence-linked endogenous biosynthetic routes.

### 5.4 Controlled photodynamic activation of porphyrin-associated pathways

Among porphyrin-based antimicrobial strategies, photodynamic therapy (PDT) currently represents the most clinically advanced and mechanistically distinctive approach. Unlike classical antibiotics or SMIs that block discrete enzymatic targets, PDT exploits endogenous porphyrin uptake and accumulation within bacterial cells to deliver light-activated cytotoxicity. Upon illumination, porphyrin-based photosensitizers generate ROS, including singlet oxygen, that inflict rapid oxidative damage on cellular membranes, proteins, nucleic acids, and secreted virulence factors (Castano et al., 2004; Amos-Tautua et al., 2019). This mechanism circumvents many conventional resistance pathways by avoiding dependence on a single molecular target.

Importantly, recent antibacterial PDT platforms increasingly utilize porphyrin-derived photosensitizers that structurally mimic endogenous bacterial porphyrins, rather than relying on non-porphyrin chromophores. This shift enhances selective uptake by pathogenic bacteria that actively accumulate porphyrins through biosynthetic “overflow,” uptake systems, or salvage pathways, while limiting accumulation in host cells (Zhang et al., 2014; Amin et al., 2016). In murine wound and infection models, porphyrin-based PDT has demonstrated robust activity against multidrug-resistant pathogens such as *P. aeruginosa* and *Acinetobacter baumannii* (*A. baumannii)*, highlighting its capacity to exploit bacterial porphyrin biology for targeted antimicrobial action (Zhang et al., 2014; Amin et al., 2016).

Beyond direct bacterial killing, PDT exerts pronounced anti-virulence effects. PDT-generated ROS rapidly oxidize extracellular enzymes, toxins, and structural components of biofilms, reducing bacterial pathogenicity even when complete eradication is not achieved (Amos-Tautua et al., 2019). This rapid functional inactivation minimizes opportunities for resistance selection and helps explain the consistently low incidence of resistance reported for PDT-based antimicrobial interventions across repeated treatments (Amos-Tautua et al., 2019). Because oxidative damage is multi-target and spatially confined to illuminated regions, bacteria face a high evolutionary barrier to developing compensatory mechanisms.

PDT also excels in contexts where conventional antibiotics perform poorly, particularly against biofilm-embedded communities. Porphyrin photosensitizers penetrate biofilm matrices and generate localized ROS that disrupt biofilm architecture while simultaneously impairing bacterial stress tolerance (Amos-Tautua et al., 2019; Amin et al., 2016). As biofilms represent a major driver of chronic infection and antimicrobial recalcitrance, this property positions PDT as a valuable addition to the antimicrobial arsenal, especially in wound, dental, and device-associated infections.

Recent studies demonstrate that PDT integrates effectively into combination and adjuvant regimens. Porphyrin-based photosensitizers do not chemically inactivate conventional antibiotics under illumination and can enhance antibiotic efficacy when used in combination, enabling faster bacterial clearance and reduced antibiotic exposure (Acosta et al., 2025). Translational platforms incorporating porphyrin mimics into wound dressings and hydrogels exemplify this potential. For example, heme-mimicking bacterial cellulose hydrogels incorporating gallium-based photosensitizers combine porphyrin-mediated targeting with PDT and chemical antimicrobial activity, achieving strong antibacterial effects and improved wound healing *in vivo* (Jiang et al., 2026).

Despite these advantages, PDT remains constrained by its reliance on light activation, limiting its utility to infections accessible to illumination, typically within approximately one centimeter of the tissue surface (Amos-Tautua et al., 2019). Additional challenges include suboptimal aqueous solubility of many porphyrin photosensitizers and the need for controlled light delivery. However, rapid advances in porphyrin chemical modification, nanocarrier formulation, and hybrid PDT/photothermal platforms are actively addressing these limitations and expanding the range of treatable infections (Amirinejad and Shiri, 2026; Koza et al., 2025; Su et al., 2026).

Collectively, PDT represents a compelling demonstration of how porphyrin biology itself can be harnessed as a therapeutic platform, rather than merely inhibited. Its pathway-selective uptake, multi-target oxidative mechanism, and compatibility with anti-virulence and adjuvant strategies position PDT as a leading example of next-generation porphyrin-based antimicrobial design. As discussed in the following section, AI-driven approaches are poised to further optimize porphyrin scaffolds, improve tissue penetration and activation profiles, and potentially enable light-independent or internally triggered variants of porphyrin-mediated antimicrobial therapy.

### 5.5 Porphyrin-targeted strategies as antimicrobial adjuvants

Beyond their standalone antimicrobial activity, porphyrin- and heme-targeted interventions are particularly well suited for use as antimicrobial adjuvants. By disrupting porphyrin-associated virulence functions, such as intracellular iron mobilization, redox buffering, and stress tolerance, these strategies weaken pathogenic fitness without directly targeting essential viability pathways. This mode of action sensitizes bacteria to existing antibiotics while avoiding the strong selective pressures typically imposed by bactericidal agents (Centola et al., 2020; Heras et al., 2015).

The adjuvant potential of porphyrin-targeted approaches is well illustrated by iron mobilization inhibitors targeting BfrB. Small-molecule inhibitors that block the BfrB–Bfd interaction in *P. aeruginosa* irreversibly sequester intracellular iron, impairing respiration and redox balance while significantly potentiating the bactericidal activity of fluoroquinolones, which inhibit DNA gyrase and topoisomerase IV, in both planktonic and biofilm-associated cells (Punchi Hewage et al., 2019; Soldano et al., 2020). This synergy highlights how selective disruption of porphyrin-associated intracellular control points can enhance antibiotic efficacy downstream of uptake, limiting compensatory responses through alternative iron-acquisition pathways (Eshelman et al., 2017).

Similar sensitizing effects have been reported for porphyrin and metal mimics. Gallium-based compounds hijack iron- and heme-uptake pathways and disable redox-dependent processes, thereby increasing bacterial susceptibility to conventional antibiotics (Centola et al., 2020). GaPPIX and related porphyrin mimics enhance intracellular delivery via heme-specific transport systems and show improved activity against biofilm-embedded and multidrug-resistant pathogens, providing a mechanistic basis for their use in combination regimens (Arivett et al., 2015; Chang et al., 2016; Hijazi et al., 2017).

PDT further extends the adjuvant concept by coupling porphyrin-mediated targeting with externally triggered oxidative stress. Porphyrin-based photosensitizers selectively accumulate in bacterial cells via endogenous porphyrin pathways and, upon light activation, generate ROS that accelerate bacterial killing and inactivate virulence factors (Amin et al., 2016; Amos-Tautua et al., 2019). When combined with conventional antibiotics, PDT has been shown to enhance antimicrobial efficacy without compromising drug stability, supporting its utility as an adjunct rather than a replacement therapy (Acosta et al., 2025). Integration of porphyrin-based PDT into multifunctional delivery platforms, including heme-mimicking hydrogels, further reinforce its translational relevance in wound and surface-associated infections (Jiang et al., 2026).

These examples demonstrate that porphyrin-targeted interventions are particularly effective in an adjuvant context. By destabilizing virulence-associated porphyrin handling, these strategies sensitize pathogens to existing antimicrobials, enable dose reduction, and reduce the evolutionary incentives for resistance. As such, porphyrin-based adjuvants represent a practical and evolutionarily informed approach to extending the clinical lifespan of current antibiotic therapies while aligning closely with anti-virulence principles (Centola et al., 2020; Heras et al., 2015).

Taken together, these therapeutic efforts reveal a consistent pattern: strategies that directly disrupt porphyrin-associated metabolism or acquisition impose substantial selective pressure and face rapid compensation, whereas approaches that modulate porphyrin-dependent virulence functions, regulatory feedback, or intracellular control points better align with anti-virulence principles. Continued innovation will be essential to overcome current limitations in tissue penetration, solubility, and delivery, and to further develop strategies that exploit porphyrin-mediated targeting to disrupt virulence while minimizing selective pressures that drive AMR. These insights point toward future therapeutic designs that combine selective porphyrin-pathway modulation with systems-level understanding of bacterial adaptation, an approach that will likely benefit from AI-guided discovery.

### 5.6 Clinical translation and current status of porphyrin-targeted anti-virulence strategies

Despite growing interest in porphyrin-centered antimicrobial strategies, no porphyrin-targeted anti-virulence therapeutic has yet received regulatory approval. Most candidate interventions remain at preclinical or early translational stages, reflecting both the complexity of porphyrin biology and the challenges associated with selectively modulating these pathways without imposing excessive selective pressure or host toxicity. Nonetheless, a diverse body of work now delineates which porphyrin-associated strategies are most likely to translate successfully and which approaches may be intrinsically limited.

A number of porphyrin-targeted compounds have demonstrated robust antimicrobial activity by directly disrupting endogenous porphyrin biosynthesis or its regulatory control. Alaremycin, a structural analogue of 5-aminolevulinic acid (ALA), competitively displaces the native precursor and suppresses porphyrin biosynthesis, resulting in growth inhibition across both Gram-positive and Gram-negative bacteria (Heinemann et al., 2010; Chen and Skaar, 2025; Okui et al., 2025). Similarly, VU0038882 lethally perturbs heme biosynthesis in Gram-positive pathogens by hyperactivating coproporphyrinogen oxidase (HemY), driving toxic accumulation of CPIII and heme (Choby and Skaar, 2016; Surdel et al., 2017). Related constraints are observed for compounds such as Xanthocillin X, which binds intracellular heme and disrupts negative feedback regulation of porphyrin biosynthesis, leading to overproduction and cytotoxic accumulation of endogenous heme in Gram-negative bacteria (Section 2.2) (Choby and Skaar, 2016; Hubner et al., 2021). Although methylated derivatives of Xanthocillin X show improved resilience against efflux-mediated resistance, host cytotoxicity remains a significant concern (Hubner et al., 2021; Shang et al., 2012).

Collectively, these biosynthesis-deregulating strategies provide valuable proof-of-principle that porphyrin pathways are pharmacologically vulnerable. However, they predominantly act through survival-targeting mechanisms, imposing strong selective pressure and engaging canonical resistance routes such as altered permeability, efflux, or bypass via increased reliance on exogenous heme acquisition. In several cases, suppressing endogenous biosynthesis may inadvertently reinforce virulence-associated uptake systems, undermining long-term therapeutic durability. As such, these approaches highlight important translational constraints rather than serving as optimal exemplars of anti-virulence design.

In contrast, porphyrin-targeted strategies that modulate virulence-linked processes, such as porphyrin acquisition hierarchy, intracellular control of iron release, redox buffering, or regulated porphyrin degradation, are better aligned with anti-virulence objectives. Interventions discussed in Sections 5.2–5.5, including inhibition of bacterioferritin-mediated iron mobilization, attenuation of heme degradation signaling, porphyrin pathway mimicry via GaPPIX, transcriptional suppression of heme uptake systems, and context-specific photodynamic activation, reduce pathogenic fitness while preserving basal viability. Importantly, these strategies tend to exploit regulatory feedback and metabolic fragility rather than collapsing core biosynthetic capacity, thereby lowering selective pressure and minimizing disruption to commensal microbiota. The porphyrin-targeted therapeutic strategies discussed in Sections 5.1–5.6 are comparatively summarized in **Table 1**, highlighting their mechanisms of action, translational status, and alignment with anti-virulence principles.

**Table 1:** Comparative summary of porphyrin-targeted therapeutic strategies showcasing anti-virulence mechanisms, with selected survival-targeting approaches included for comparison.

Targeted pathway	Strategy	Representative compound(s)	Advantages	Limitations	Development stage
Metal uptake	Chelation	^†^EDTA (Repac Antic et al., 2022); ^†^DFO (Moon et al., 2011); ^†^Nitroxoline (Repac Antic et al., 2022)	Broad antimicrobial activity; inhibits adhesion and biofilm formation	Non-specific metal binding; disrupts host and commensal metal homeostasis; survival-targeting increases risk of AMR	Not yet approved (EDTA, DFO); country-specific approval (Nitroxoline)
	Activation of uptake	^†^VU002692 (Juttukonda et al., 2020)	Potent bactericidal activity	Non-specific; host toxicity risk; survival-targeting increases risk of AMR	Discovery and development
	Siderophore-mediated antibiotic delivery	^†^Cefiderocol (Repac Antic et al., 2022)	Potent bactericidal activity; effective against MDR Gram-negative pathogens	Broad-spectrum; survival-targeting increases risk of AMR	Approved
Iron storage	Inhibition of BfrB-Bfd interaction	4-aminoisoindoline-1,3-dione analogues (Punchi Hewage et al., 2019)	Virulence-linked mechanism minimizes risk of AMR; suppresses biofilms; predicted low host toxicity; synergy with combinational therapies	Intracellular targeting increases risk of efflux/influx-based resistance; unknown PK/toxicity	Discovery and development
Heme degradation	HemO inhibition	Small-molecule inhibitors (Liang et al., 2018; Hom et al., 2013); Iminoguanidine-based allosteric inhibitors (Heinzl et al., 2016)	Virulence-targeting minimizes the risk of AMR; predicted low host toxicity; reduces heme uptake via feedback	Intracellular targeting increases risk of efflux/influx-based resistance; unknown PK/toxicity	Discovery and development
Heme biosynthesis deregulation	Biosynthesis inhibition	^†^Alaremycin (Okui et al., 2025)	Potent bactericidal activity; broad activity; blocks endogenous porphyrin production	Broad-spectrum; survival-targeting increases risk of AMR; intracellular targeting increases risk of efflux/influx-based resistance	Preclinical
	Feedback disruption/Biosynthesis hyperactivation	^†^Xanthocillin X (Hubner et al., 2021; Shang et al., 2012)	Potent bactericidal activity; broad activity; induces toxic heme overaccumulation; efflux-resistant derivatives available	Broad-spectrum; survival-targeting increases risk of AMR; intracellular targeting increases risk of efflux/influx-based resistance; cytotoxicity in human cells;	Preclinical
	Enzyme hyperactivation	^†^VU0038882 (Surdel et al., 2017)	Selective HemY activation; predicted low host toxicity	Survival-targeting increases risk of AMR; Intracellular targeting increases risk of efflux/influx-based resistance; selective range not exclusive of commensal bacteria	Preclinical
Porphyrin Acquisition	Transcriptional modulation	^‡^2R,3R-dihydromyricetin (Ran et al., 2023b)	Potent bactericidal activity; anti-virulence effects (inhibits heme acquisition, anti-biofilm); inhibits biofilm formation; multi-system targeting minimizes the risk of AMR	Survival-targeting increases risk of AMR; intracellular targeting increases risk of efflux/influx-based resistance;	Preclinical
	Hemophore inhibition (Gram-positive)	VHH antibodies (IsdH/IsdB) (Valenciano-Bellido et al., 2023); IsdB small-molecule inhibitors (Cozzi et al., 2024)	Virulence-targeting minimizes the risk of AMR; extracellular mechanism not subject to efflux/influx-based resistance; selectively targets gram-positive pathogens; active against MRSA	Unknown PK/toxicity	Discovery and development
	Hemophore inhibition (Gram-negative)	Fe-phthalocyanine; Fe-salophen (Baecker et al., 2021; Shirataki et al., 2014)	Virulence-targeting minimizes the risk of AMR; tunable specificity; extracellular mechanism not subject to efflux/influx-based resistance; selectively targets gram-negative pathogens; synergy with combinational therapies; enables intracellular delivery of conjugated antimicrobials; modulates heme-responsive regulation	Unknown PK/toxicity	Discovery and development
	Metal mimicry	^‡^Gallium nitrate (Goss et al., 2018)	Low resistance propensity; minimal host toxicity	Broad-spectrum; limited pathogen specificity	Clinical Phase I
	Porphyrin-conjugated metal mimicry	GaPPIX (Hijazi et al., 2017); GaMPIX (Hijazi et al., 2017)	Virulence-targeting lowers the risk of AMR; enhanced activity vs MDR and biofilms; tunable specificity: synergy with combinational therapies; enables intracellular delivery of conjugated antimicrobials	Cytotoxicity at high doses; incomplete eradication risk despite bactericidal properties increases the risk of AMR	Preclinical
	Porphyrin-based photodynamic therapy (PDT)	Porphyrin-AgNPs conjugates (Elashnikov et al., 2019); TFPP-QA/CP5 (Xia et al., 2022); mPL-TCPP (Ran et al., 2023a); PHEMA-Porph (Castro et al., 2019)	Rapid action; multi-system targeting effect; low resistance emergence; high, tunable specificity; strong anti-virulence effects (degrades extracellular enzymes, toxins, and biofilm structural components); limited host toxicity; synergy with combinational therapies	Light penetration is limited to ~1 cm; ineffective against deep-seated and systemic infections; commonly exhibits poor solubility	Discovery and development to Preclinical
	Delivery platforms for PDT	Porphyrin hydrogels (Jiang et al., 2026); porphyrin-nanocarriers (Amirinejad and Shiri, 2026)	Enhanced localization; improved solubility; controlled activation; effective in wound/biofilm contexts	Light penetration is limited to ~1 cm; ineffective against deep-seated and systemic infections; local use	Discovery and development to Preclinical

AgNPs, silver nanoparticles; AMR, antimicrobial resistance; Bfd, bacterioferritin-associated ferredoxin; BfrB, bacterioferritin B; CPIII, coproporphyrin III; DFO, deferoxamine; HemO, heme oxygenase; MDR, multidrug-resistant; PDT, photodynamic therapy; PK, pharmacokinetics.

†Primarily survival-targeting strategies included for contextual comparison. These approaches inhibit bacterial growth or viability and generally impose stronger selective pressure for antimicrobial resistance than anti-virulence-aligned strategies.

‡Compounds exhibiting mixed or context-dependent activity, combining virulence modulation with significant effects on bacterial growth or viability.

From a translational perspective, porphyrin-targeted anti-virulence strategies are most likely to gain clinical traction in defined contexts where modulation rather than eradication is advantageous, such as chronic infections, biofilm-associated disease, polymicrobial dysbiosis, and adjuvant therapy to enhance antibiotic efficacy. As with other anti-virulence strategies, these approaches are expected to be most effective when combined with host immunity or conventional antimicrobials, rather than as standalone curative agents. Continued innovation will be essential to overcome current limitations in tissue penetration, solubility, and delivery, and to further refine strategies that exploit porphyrin-mediated targeting to disrupt virulence while minimizing selective pressures that drive AMR. These translational challenges and opportunities naturally motivate the application of AI-guided discovery and optimization frameworks, discussed in the following section, to systematically prioritize targets and design porphyrin-based interventions with improved selectivity, pharmacological properties, and durability.

## 6 AI opportunities for targeting heme pathway proteins

Heme synthesis and acquisition in bacteria is mediated by intricate and diverse systems (Choby and Skaar, 2016). Many of the protein components in such systems are experimentally characterized in model organisms. From a drug discovery perspective, these proteins are druggable yet difficult owing to a conglomerate of challenges. First, porphyrin-binding pockets are significantly hydrophobic, which affects the downstream drug discovery efforts by conflicting with standard rules for developing conventional drug-like inhibitors. Such hydrophobicity also creates challenges for solubility and delivery of potential inhibitors. The lack of hydrogen bonds or electrostatic interactions negatively affect the selectivity of the potential inhibitor. Moreover, these pockets are evolutionary conserved, therefore heme mimetics are generally unfavored due to potential off-target binding and host toxicity (Chen and Skaar, 2025). Second, pocket accessibility is another challenge, especially in heme sensor proteins such as DosS in *Mycobacterium tuberculosis* in which heme is enclosed within the GAF domain (Cho et al., 2009). Third, heme binding in such diverse pockets is not identical. This diversity in binding modes is reflected by iron atom coordination by histidine residue with either tyrosine or a water molecule, and the overall orientation of heme that could vary between heme-binding proteins. Fourth, due to the paramount importance of heme, bacteria have developed redundant pathways for heme synthesis and acquisition (Choby and Skaar, 2016). Highly specific inhibition of a protein within the heme synthesis chain to limit an intermediate product has less chance for success since such product can be compensated via an alternative source(s).

Since targeting porphyrin pathways is anti-virulence strategy in its core, such approach also has additional criteria that differ from the conventional antibiotic discovery. Anti-virulence approach aims at disarming the pathogens from the virulence factor while allowing cell viability to minimize the selective pressure and consequently the development of resistance. This necessitates careful selection of protein targets involved in non-essential processes such as heme acquisition, biofilm formation, toxins production, and quorum sensing (Heras et al., 2015). As a consequence to these requirements and challenges, designing heme/porphyrin-targeting agents requires navigating an enormous chemical and biological design space that ranges from metal binding, redox behavior, hydrophobicity, and transporters to pathogen-specific targets, efflux, membrane barriers, and compensatory metabolic networks. AI excels in such scenarios because deep generative and high dimensional models can search large datasets of molecular and chemical properties to uncover feature combinations that are beyond human capabilities.

### 6.1 AI-guided structure-based drug discovery

**Figure 7:**
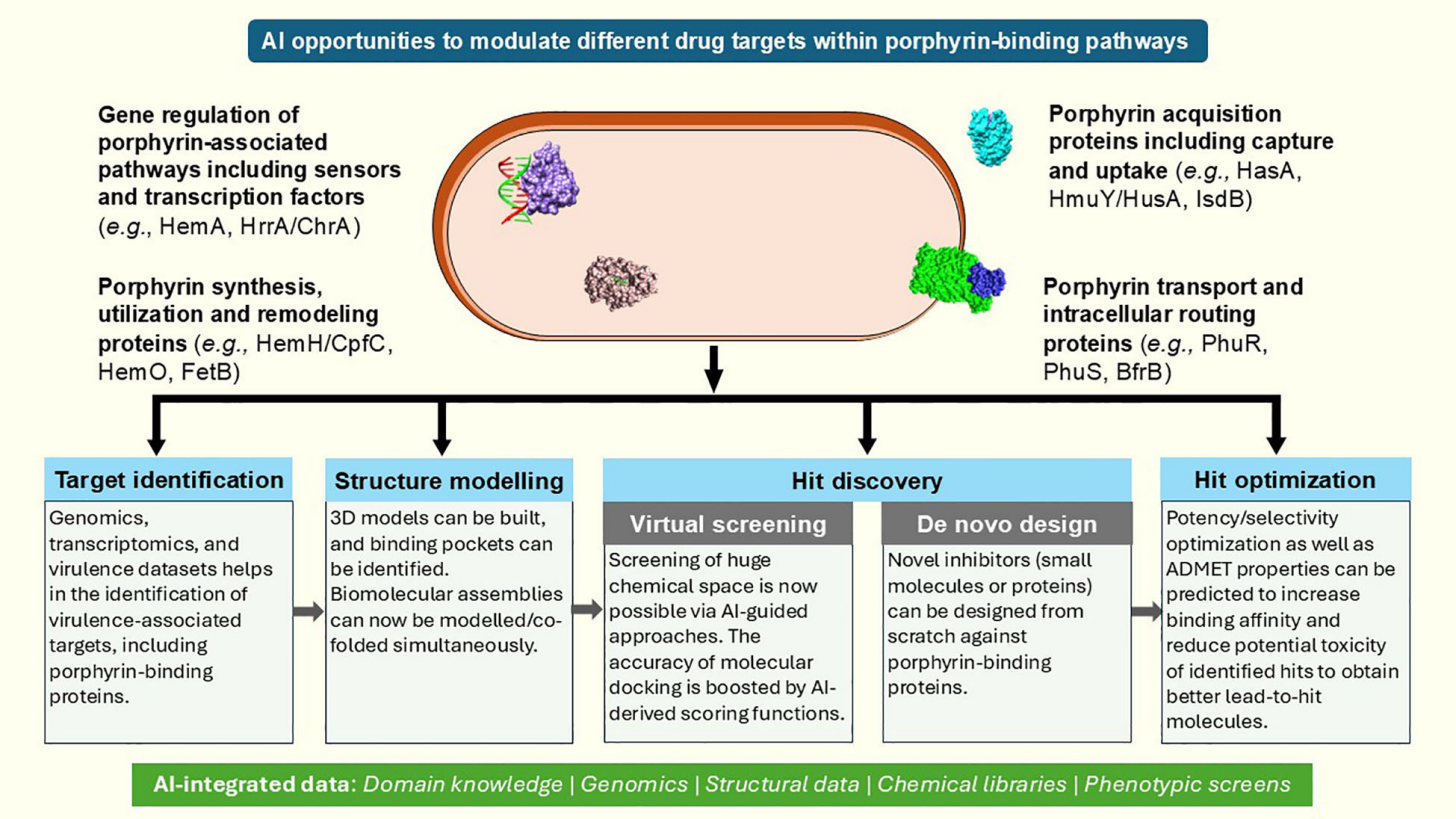
AI-enabled discovery and optimization of porphyrin-targeted anti-virulence strategies. Schematic illustrating how AI and ML, machine learning approaches can be applied across the full discovery pipeline for porphyrin-associated antimicrobial targets. Multi-omic, including genomics, transcriptomics, and virulence-associated datasets, support target identification of porphyrin-binding proteins involved in gene regulation, acquisition, transport, synthesis, and utilization. Advances in structure modelling enable prediction of three-dimensional protein structures, identification of porphyrin-binding pockets, and co-folding of biomolecular assemblies. AI-guided virtual screening allows rapid exploration of large chemical libraries using enhanced docking and scoring functions. *De novo* design frameworks facilitate the generation of small-molecule inhibitors or protein binders against porphyrin-associated targets, followed by hit optimization to improve potency, selectivity, and pharmacokinetic (including absorption, distribution, metabolism, excretion, and toxicity) properties.

AI has transformed science and research, including drug discovery campaigns. Advances in deep learning (DL) have undoubtedly enhanced biomolecular structure prediction, cryptic pocket identification, virtual screening, toxicity prediction, and the *de novo* design of binders and small-molecule inhibitors (Zhang et al., 2025; Abduljalil et al., 2026). **Figure 7** summarizes how AI-guided approaches can be applied for drug discovery purposes across multiple layers of porphyrin-associated biology, from gene regulation and acquisition to intracellular processing and transport. Collectively, these capabilities facilitate the discovery of modulators or inhibitors for targets that were previously regarded as undruggable or extremely difficult to modulate. AI-guided identification of pathogen-specific allosteric pockets on porphyrin-binding proteins might bypass most of the challenges mentioned previously, especially if the allosteric pocket contains hydrophobic and hydrophilic regions. SMIs have been reported for some heme-binding proteins, including HemO in *P. aeruginosa* and *Neisseria meningitidis*, and HemY in *S. aureus* (Furci et al., 2007; Lobo et al., 2015). Although these molecules can be used as query for AI-guided ligand-based virtual screening, this approach is more fruitful in finding better or more potent binders than to identify the first known inhibitors. On the contrary, AI-guided structure-based virtual screening and molecular docking has enabled rapid and prioritization of potential inhibitory compounds from ultra-large chemical libraries, significantly reducing the experimental costs and improving hit discovery efficiency (Lluka and Stokes, 2023). A recently published docking model, MetalloDock, outperformed classical docking programs in docking small molecules into metalloprotein pockets in terms of enrichment factor (top 0.5–1%) and docking success rate (RMSD 2 Å with chemically and physically valid poses) (Zhang et al., 2026). Additionally, a recent benchmarking of AI-guided molecular docking tools (Gu et al., 2025) and AlphaFold3-like models (Shen et al., 2026) has shown promising performance over the physics-based tools in terms of identifying true active inhibitors. Such capabilities are expected to aid in discovery of potential orthosteric or allosteric inhibitors against bacterial heme-binding proteins (Heinzl et al., 2016). However, many of these tools remain impractical for large-scale virtual screening because prediction takes too long (minutes/molecules). Additionally, many porphyrin-binding proteins exhibit pronounced structural plasticity that further complicates inhibitor design. While some heme-binding enzymes, such as HemO oxygenases, contain relatively well-defined binding pockets, other porphyrin-binding proteins such as extracellular hemophores adopt highly flexible, induced-fit conformations during heme binding and transfer (*e.g.*, HasA in *S. marcescens* and *P. aeruginosa*, HusA in *P. gingivalis*, and HphA in *A. baumannii*). This conformational variability challenges conventional structure-based drug discovery approaches that assume static pockets, reinforcing the need for methods capable of efficiently capturing protein dynamics and transient binding states. While capturing such conformational ensembles and transient binding states lies beyond the scope of conventional docking, they are within the emerging capabilities of AI-guided and dynamics-aware discovery pipelines.

### 6.2 AI-driven *de novo* binder design

Although the classical approaches of structure-based drug design showed some success in inhibiting certain porphyrin-binding proteins (Liang et al., 2018), the inhibitors were designed by human experts through fusing benzene derivatives to a thioxothiazolidin-propanoate scaffold to mimic > 50% of heme structure. However, toxicity of these inhibitors in eukaryotic cells is anticipated, yet still unclear. Structure-aware generative diffusion models opened new opportunities via *de novo* design of inhibitors and modulators tailored to a user-specified target protein structure (Crucitti et al., 2024). Such capability not only increases the chance of developing effective inhibitors but also produces chemically novel scaffolds (Ozcelik et al., 2025) with acceptable drug-like properties (Schneuing et al., 2024; Peng et al., 2022). Despite their success in different cases, generative models still struggle with generating binding poses with chemical and physical plausibility (Harris et al., 2023). Similarly, DL-based protein design approaches enabled the construction of high-affinity binders (Fox et al., 2025b).

A landmark diffusion-based application for a porphyrin-associated virulence system was demonstrated by the development of a potent antibacterial inhibitor of the outer membrane transporter ChuA that hijacks heme from host hemoglobin (Fox et al., 2025a). The *de novo* design started by using an AlphaFold model of ChuA as a target receptor to which binders were generated. ChuA is a heme transporter that targets dimeric hemoglobin (a high-affinity substrate) and myoglobin (a low-affinity substrate). The design process was directed toward the flexible extracellular region of ChuA, specifically loops 7 and 8, which are responsible for heme binding and extraction. A total of 20,000 binders were designed computationally and filtered down to 96 candidates for experimental testing. The initial screening via spotting on bacterial lawn on agar plates identified promising binders characterized by distinct inhibition zones. The diameters of these zones were inversely proportional to hemoglobin concentrations, indicating a competitive mechanism. Furthermore, free hemin transport remained unaffected by the binders, suggesting that they inhibit heme extraction rather than the transport function of ChuA itself. Potency (IC_50_ ranging from 42.5 nM to 3.3 µM) and affinity (K_d_ ranging from 64 nM to 155 nM) were determined for the three leading binders. When the solved structures of the ChuA:binder complexes were superimposed onto the computational models, the RMSD values for non-flexible regions were < 1 Å. This study demonstrates that diffusion-based protein design can produce functional inhibitors that closely match experimental results with minimal optimization.

More broadly, this study demonstrates that diffusion-based protein design can effectively engage porphyrin-associated uptake systems characterized by hydrophobic ligand environments, metal-coordinating chemistry, and conformationally dynamic binding regions, features that have historically limited traditional small-molecule inhibitor discovery. The ability of AI-guided workflows to rapidly sample large conformational and sequence spaces allows prioritization of a small number of high-quality candidates for experimental testing within weeks to months, compared to the years often required by iterative, manually driven traditional approaches. The high apparent hit rate observed for ChuA further suggests that AI-designed binders are particularly well suited to targets presenting exposed loops or spatially projecting features that can be sterically occluded by designed proteins.

While the ChuA binders illustrate the power of AI-driven protein design for porphyrin-associated virulence systems, translational challenges remain. As with other protein-based therapeutic modalities, questions surrounding immunogenicity, *in vivo* stability, delivery, and manufacturability require careful evaluation. Nonetheless, this work provides a compelling proof-of-principle that AI-guided binder design can achieve functional inhibition of porphyrin-linked uptake processes with high structural fidelity, establishing a foundation for broader application of AI to porphyrin-centered anti-virulence strategies discussed throughout this review.

### 6.3 AI-guided optimization from hit to lead

Once an initial hit has been identified, DL can be harnessed for multi-objective hit-to-lead optimization. This process improves not only the binding affinity, but also to increase selectivity to minimize the unwanted effects on the host and the non-pathogenic microbiota (Sun et al., 2022). High selectivity can be achieved by exploiting the subtle changes in target structures and differences in metal-binding geometry. This would lead to a pocket-tailored narrow-spectrum inhibitor with a reduced off-target binding as porphyrin-binding pathways are considerably conserved. Different models have been developed and successfully improved the initial hits (Zhang et al., 2024; Fang et al., 2025). Moreover, generative AI has opened new opportunities for scaffold hopping in which the core of an active molecule is replaced by a new structurally different core while retaining the groups at the periphery that interact with the receptor (Zhang et al., 2024). This would significantly enhance the selectivity and delivery of inhibitors designed against a specific set of heme-binding proteins that share pocket architecture and heme-binding mode.

## 7 Conclusions and future directions

### 7.1 Key advances and remaining challenges

Porphyrin-centered biology has emerged as a critical, yet underexploited, axis of bacterial pathogenic fitness. Across diverse pathogens, porphyrins and heme-associated pathways integrate energy metabolism, redox regulation, metal homeostasis, and environmental sensing, functions that are repeatedly co-opted to support virulence, stress tolerance, and persistence within host niches. The work surveyed in this review demonstrates that porphyrins are not merely metabolic cofactors but dynamic biological signals and structural modulators that shape host-pathogen interactions, polymicrobial community behavior, and infection outcomes.

**Figure 8:**
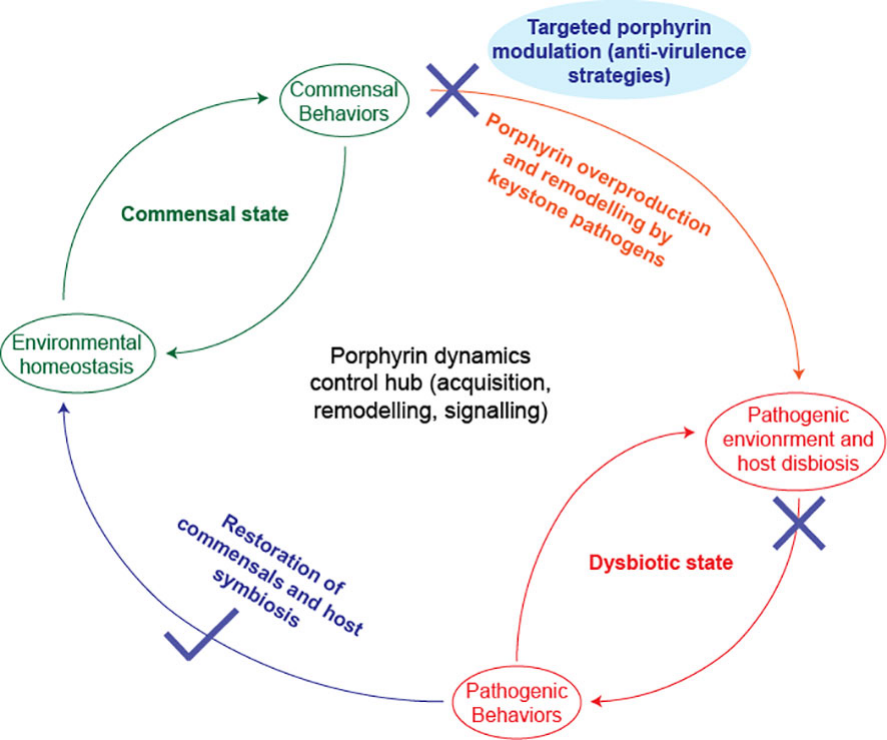
Porphyrin dynamics as a central control hub governing commensal-pathogenic transitions. Porphyrin dynamics, including acquisition, remodeling, and signaling, operate as a central control hub linking microbial behaviors, environmental state, and host-microbiota interactions. Under commensal conditions (green), balanced porphyrin handling supports environmental homeostasis and stable commensal behaviors. In contrast, porphyrin overproduction and remodeling driven by keystone pathogens (e.g., manganese protoporphyrin IX formation) shifts porphyrin availability and redox balance, promoting dysbiotic environments and pathogenic behaviors (red). These pathogenic states reinforce each other, sustaining community disruption and host dysbiosis. Targeted modulation of porphyrin-associated pathways (blue) represents an anti-virulence strategy that selectively interrupts this trajectory, enabling restoration of commensal populations, host symbiosis, and environmental homeostasis without directly targeting bacterial viability.

Historically, antimicrobial strategies targeting metals or iron metabolism struggled due to poor specificity, host toxicity, and rapid bacterial compensation. However, recent advances reveal that selectively targeting porphyrin-dependent processes, particularly those linked to acquisition, remodeling, and regulated degradation, offers a fundamentally different therapeutic logic. Rather than eliminating bacterial viability, these approaches attenuate pathogenic potential, destabilize virulence-supporting microenvironments, and weaken cooperative behaviors that underlie chronic and polymicrobial infections. Importantly, this anti-virulence framework reframes failure not as incomplete killing, but as deliberate disruption of pathogenic fitness (**Figure 8**).

Despite this progress, significant challenges remain. Porphyrin and heme pathways are intrinsically redundant, dynamically regulated, and often conserved across taxa, complicating efforts to achieve selectivity without off-target effects. Intracellular targets are vulnerable to classical resistance mechanisms such as reduced permeability and efflux, while extracellular strategies risk compensatory shifts toward alternative acquisition routes. These limitations underscore the importance of pathway-level thinking and rational target prioritization rather than isolated target inhibition.

### 7.2 Virulence-focused antimicrobials as the future of AMR combat

The evidence assembled in this review supports a central conclusion: porphyrin-centered pathways exemplify how anti-virulence strategies can decouple pathogenicity from survival, thereby reducing the selective pressures that drive AMR. By focusing on metabolic and regulatory nodes that are essential for infection but dispensable for basal viability, porphyrin-targeted interventions offer a means to reshape bacterial evolution toward lower virulence states.

This approach is particularly powerful in polymicrobial contexts, where porphyrin dynamics promote cooperative virulence, immune modulation, and dysbiosis. Disrupting shared porphyrin pools, acquisition hierarchies, or remodeling mechanisms has the potential to destabilize entire pathogenic communities by targeting keystone species or shared dependencies. Such strategies may restore ecological balance, expose pathogens to host immunity, and improve the efficacy of existing antibiotics without exerting strong bactericidal pressure.

Future therapeutic development is therefore likely to benefit from combination strategies that integrate porphyrin-targeted anti-virulence agents with traditional antimicrobials. By weakening bacterial defenses, limiting stress tolerance, or blocking compensatory pathways, porphyrin-based interventions can sensitize pathogens to lower doses of conventional antibiotics, extending their clinical lifespan while mitigating resistance emergence.

### 7.3 The AI-accelerated future

Perhaps the most promising development in this field is the convergence of porphyrin biology with AI-enabled drug discovery. Heme-binding proteins present challenges that have historically limited structure-based drug design, including hydrophobic pockets, metal coordination chemistry, dynamic binding modes, and pathway redundancy. AI-driven approaches are uniquely suited to navigate this complexity.

Recent advances in structure prediction, metalloprotein-specific docking, generative chemistry, and multi-objective optimization enable the rational design of ligands and binders that balance affinity, selectivity, toxicity avoidance, and delivery constraints. Importantly, AI does not merely accelerate discovery, it enables a shift from single-target inhibition toward network-aware design, allowing simultaneous consideration of compensatory pathways and anti-virulence objectives.

Looking forward, the most successful porphyrin-targeted therapies are likely to arise from integrative pipelines that combine biological insight, structural data, evolutionary reasoning, and AI-guided optimization. Such approaches can prioritize targets based on virulence relevance rather than essentiality, identify synergistic target combinations, and tailor interventions to pathogen-specific porphyrin dependencies.

### 7.4 Outlook

In summary, porphyrin-centered biology represents a conceptual and practical bridge between bacterial physiology, virulence, and therapeutic innovation. By reframing porphyrins as modulators of pathogenic behavior rather than mere metabolic intermediates, new opportunities emerge for combating AMR through precision anti-virulence strategies. As AI-driven discovery continues to mature, the longstanding challenges of targeting porphyrin and heme pathways are becoming tractable, paving the way toward first-in-class antimicrobials that disrupt infection without accelerating resistance.
